# Conformational changes induced by K949A mutation in the CRISPR-Cas12a complex drives an effective target-binding mechanism

**DOI:** 10.1016/j.crstbi.2025.100173

**Published:** 2025-08-08

**Authors:** Pragya Kesarwani, Durai Sundar

**Affiliations:** a Regional Centre for Biotechnology, Faridabad, 121001, Haryana, India; b Department of Biochemical Engineering and Biotechnology, Indian Institute of Technology (IIT) Delhi, New Delhi, 110016, India; c Yardi School of Artificial Intelligence, Indian Institute of Technology (IIT) Delhi, New Delhi, 110016, India; d Institute of Bioinformatics and Applied Biotechnology (IBAB), Bengaluru, 560100, India

**Keywords:** CRISPR-Cas12a system, Genome editing, gRNA-DNA hybrid, PAM variants, Gaussian accelerated MD simulation, Structural insights

## Abstract

The CRISPR/Cas system is a potential tool for genome editing, yet it faces challenges due to off-target activity caused by mismatches at specific positions. However, Off-target activity can be minimized by optimal design of guide RNA (gRNA) but there remains a possibility of unintended cleavage, highlighting the role of the Cas nuclease in off-target recognition and binding the target site. This study focuses on comparing the conformational dynamics and stability of Wildtype, RR, RVR, RRm and RVRm variants of AsCas12a with gRNA-DNA bound complexes. It was found that the cross-correlation coefficient between His1167 of the NUC domain and Thr384 of the REC II domain significantly increased after the K949A mutation compared to other variants. The extensive spread of principal components also revealed flexibility in both Cas nuclease and gRNA-DNA hybrid of RVR variant and wildtype AsCas12a whereas the confined clusters in PCA plot suggests increased stability in both the variants after mutation. This study shows the role of K949A mutation in improving stability of PAM variants and predicted critical residues such as His1167, Thr384 and Ser959, in inducing stability in mutants of PAM variants.

## Graphical abstract

**Figure:**
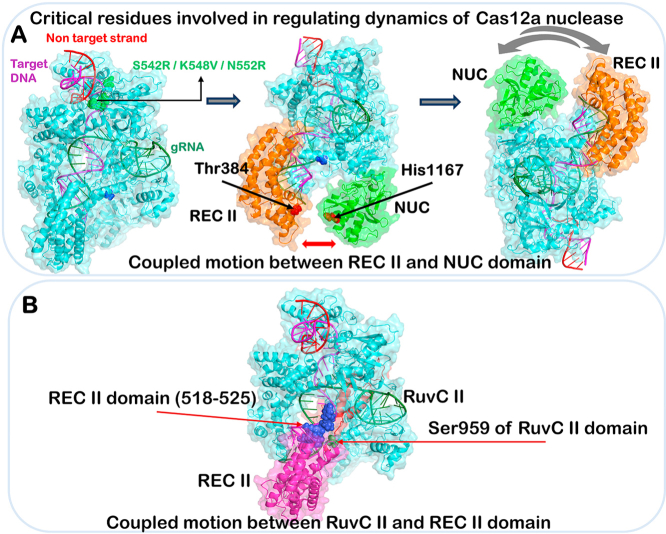

## 1 Introduction

The CRISPR/Cas system is the adaptive immune system of bacteria and archaea against phages and viral attacks. It consists of short repeats interspersed with unique spacers within the bacterial genome (Doudna and Charpentier, 2014; Horvath and Barrangou, 2010; Wang et al., 2016; Mohanraju et al., 2016; Sorek et al., 2013; Wiedenheft et al., 2012; Jinek et al., 2012). The repeat arrays undergo transcription to produce lengthy transcripts known as CRISPR RNAs for recognizing and targeting foreign DNA. These transcripts undergo processing to generate shorter CRISPR RNA (crRNAs) consisting of a spacer sequence and a segment of the neighbouring direct repeat to defend themselves from future phases of attack (Horvath and Barrangou, 2010; Gasiunas et al., 2012). The region of the target sequence closest to the PAM is referred to as the PAM proximal region, while the region farthest from the PAM is termed the PAM distal region. These regions play a crucial role in guiding RNA-DNA hybridization and determining cleavage efficiency and specificity of Cas nucleases. Recently, researchers have repurposed CRISPR/Cas system to address various genome editing applications (Cong et al., 2013; Hsu et al., 2014; Barrangou and Doudna, 2016; Tak et al., 2017). The CRISPR/Cas system utilizes a 23-nucleotide guide RNA (gRNA) to target a desired DNA sequence in a host genome. The gRNA selectively bind to the target location but on-target and off-target activities of gRNAs vary considerably due to mismatch tolerance (Kim et al., 2018, Kim et al., 2017, Kim et al., 2016; Kleinstiver et al., 2016; Zhu and Liang, 2019; Kesarwani et al., 2023). The protospacer adjacent motif (PAM) sequence near the cleavage site facilitates the clinical application of the CRISPR-Cas12a system and directs target site recognition in the host genome (Swarts and Jinek, 2018; Sun et al., 2023). Recently, Cas12a has been gaining interest due to its high on-target efficiency and low off-target activity (Zetsche et al., 2015). Various characteristic features of Cas12a include recognizing an AT-rich PAM sequence (5′-YTTN-3′), a preference for longer protospacers and the ability to generate staggered cuts distal to the PAM.

In contrast to Cas12a, Cas9 recognizes NGG PAM sequence with a protospacer limited to 20 bp and the ability to induce blunt cuts proximal to the PAM(Gasiunas et al., 2012; Jiang and Doudna, 2017). Unlike Cas9, Cas12a does not require a transactivating crRNA for nuclease activity or guide RNA (gRNA) maturation. Although Cas12a is reportedly more specific than Cas9, the kinetics and mechanism of DNA recognition and cleavage by Cas12a in response to mismatches between the guide RNA and target DNA are poorly understood (Sun et al., 2023; Anders et al., 2014; Gong et al., 2018; Jiang et al., 2016; Jinek et al., 2014; Palermo et al., 2017). Both Cas9 and Cas12a share a two-step target identification mechanism involving the recognition of PAM followed by reversible RNA-DNA heteroduplex extension. Cas12a exhibits stable binding of a 17-bp PAM-proximal match with gRNA for target cleavage, whereas Cas9 requires only 9-bp to 16-bp PAM proximal matches for stable binding and cleavage of the target sequence (Swarts and Jinek, 2018, 2019; Jiang et al., 2016; Liu et al., 2022; Jeon et al., 2018; Kim et al., 2020; Saha et al., 2020; Singh et al., 2018; Stella et al., 2018; Sternberg et al., 2014, 2015; Swarts et al., 2017; Szczelkun et al., 2014).

**Fig. 1:**
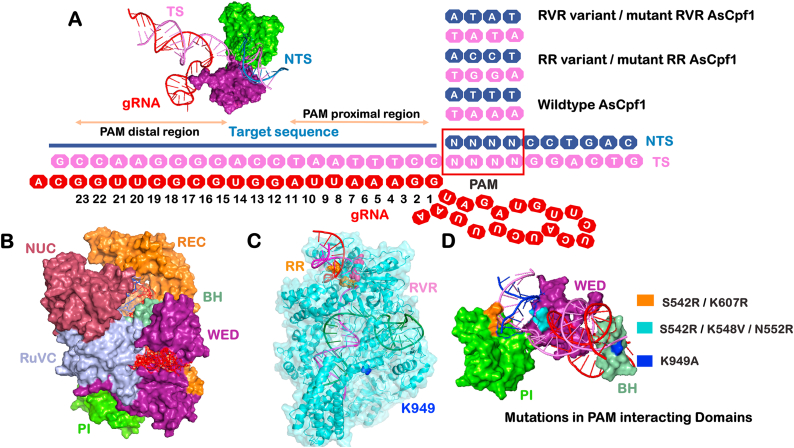
**Representation of domains and mutations in variants of AsCas12a**. (A) The sequence representation of gRNA and target DNA hybrid in the CRISPR-Cas12a system indicates the tolerance of different PAM sites in Wildtype, RR, and RVR variants of AsCas12a. (B) The crystal structure of the Cas12a extracted from *Acidaminococcus* sp. Comprises AsCas12a nuclease complexed with guide RNA (gRNA), target DNA (TS), and non-target strands (NTS). A surface representation of the AsCas12a structure depicts different domains in different colors. (C) The location of K949A, S542R, K607R, K548V and N552R mutations in the PDB structure of Cas12a nuclease. (D) The AsCas12a model system represents mutations in the PAM-Interacting (PI) domain, Bridge helix (BH), and WED domain of respective variants.

The AsCas12a protein extracted from the *Acidaminococcus* sp. Consists of 1307 amino acids. The crystal structure of AsCas12a consists of two major lobes named a nuclease lobe (NUC) and an alpha-helical recognition lobe (REC)(Dong et al., 2016; Yamano et al., 2016). The REC lobe comprises two domains named REC1 and REC2, while the NUC lobe consists of the RuvC domain and three additional domains, PI, WED, and BH. The RuvC domain in Cas12a is present in three discontinuous segments abbreviated as RuvC I–III. These domains play a specific role in inducing double-strand breaks by Cas12a. The structure of Cas9 comprises two distinct nuclease domains, namely HNH and RuvC, whereas Cas12a lacks the HNH domain and possesses a RuvC-like endonuclease domain along with a Nuc domain for DNA cleavage. Fig. 1(B) shows surface representations of domain organization in the Wildtype AsCas12a-gRNA-DNA complex. Initially, Cas12a diffuses along a DNA sequence by utilizing one-dimensional (1D) diffusion for target searching and stalls at the desired target site (Losito et al., 2021). The stalling of Cas12a at the target site leads to the cleavage of DNA fragments at the tethered sites (Singh et al., 2018). Moreover, previous studies have demonstrated that AsCas12a RNPs promptly releases the PAM distal portion of the DNA strand but continue to retain the PAM-proximal portion of the DNA strand after inducing DNA cleavage (Jeon et al., 2018; Strohkendl et al., 2018). This mechanism contrasts with SpCas9, which maintains both DNA fragments for an extended duration.

The commonly used *Acidaminococcus* sp. BV3L6 Cas12a (AsCas12a) and *Lachnospiraceae bacterium* ND2006 Cas12a (LbCas12a) are limited in utility due to their requirement for a TTTV protospacer adjacent motif (PAM) in the DNA substrate. The ‘V' in the PAM sequence represents A, C, or G whereas ‘Y' denotes a pyrimidine (C or T), ‘N' indicates any nucleotide (A, T, G, or C). Additionally, AsCas12a may sometimes recognize sequences with non-canonical C-rich PAMs (such as CTTA, TCTA, and TTCA) in mammalian cells. However, their efficiencies in cleaving these Cas12a orthologs with non-canonical PAMs are lower than those with canonical TTTV PAMs. A study was conducted on screening structure-guided mutagenesis aimed at expanding the targeting range of Cas12a to include C-rich and A-rich PAM sites. The study generated two AsCas12a variants with mutations at the S542R/K607R (RR variant) and S542R/K548 V/N552R (RVR variant) sites (Gao et al., 2017). The RR variant recognizes TYCV PAM sites and the RVR variant recognizes TATV PAM sites. The study has reported that these variants exhibit enhanced activities *in vitro* and in human cell lines. The analysis of genome-wide specificity of off-target activity using BLISS7 indicated that these variants maintain high DNA-targeting specificity (Gao et al., 2017; Nishimasu et al., 2017). The additional non-PAM interacting mutation at K949A induced enhanced activity at the target site and reduced activity at off-target locations. These variants expanded the targeting range of Cas12a approximately threefold in the human genome (Gao et al., 2017). Various computational studies exist to understand the conformational dynamics of the CRISPR/Cas system (Saha et al., 2020, Saha et al., 2024; Swarts et al., 2017; Vora et al., 2023; Ricci et al., 2019; Ray and Di Felice, 2020; Strohkendl et al., 2024). However, this study focuses on understanding the change in conformational dynamics of PAM variants of AsCas12a in compared to Wildtype AsCas12a. The domain-wise flexibility and stability of variants and their bound gRNA-DNA hybrids has shed light on the significance of mutations in driving the target specificity of AsCas12a. Understanding the sub-molecular-level interactions in variants in a cell-like environment is critical for evaluating target binding efficiency.

## 2 Results and discussion

The complementarity between guide RNA and target DNA is essential for the cleavage activity of AsCas12a. Different studies have revealed the ability of the CRISPR-Cas12a system to tolerate mismatches at specific positions leads to off-target activity (Kim et al., 2016, Kim et al., 2017; Kleinstiver et al., 2016). However, there remains a possibility of unintended cleavage indicating the role of the Cas nuclease in off-target recognition and binding. This off-target activity is also known to be influenced by the recognition mechanism and cleavage dynamics of the CRISPR/Cas system. Furthermore, the applicability of Cas12a is restricted to certain genes due to the requirement of TTTV PAM in the target sequence. The Cas12a variants selected in this study target different PAM sites (TATA, TCTY, and TTTN). Therefore, this study majorly focuses on comparing the structural stability of the gRNA-DNA hybrid among different AsCas12a PAM variants and predict critical residues for ensuring high on-target efficiency at both canonical and non-canonical PAM sites. In this study, Gaussian accelerated molecular dynamics simulations were conducted to explore the change in conformational dynamics of different domains of AsCas12a variants and stability of their gRNA-DNA hybrid.

### 2.1 Domain-specific stability in AsCas12a variants

The Cas nuclease undergoes significant conformational changes upon binding of guide RNA with target DNA with perfect complementarity and leads to the activation of cleavage activity. Therefore, this study investigated the stability of the AsCas12a-gRNA-DNA complex to highlight the difference in conformational changes between different PAM variants. The effect of mutations in inducing structural stability of the variants of the AsCas12a and their gRNA-DNA hybrid through extensive molecular dynamics simulations was studied. The NPT-equilibrated structure was used as a reference for RMSD calculations for all the AsCas12a variants. Root mean square deviation calculations revealed that the dynamic stability of all four variants and Wildtype AsCas12a was converged over time, with fluctuations ranging between 0.5 Å to 2.5 Å beyond 500 ns as summarised in Table 1. There were variations in different domains including REC, RuvC, and NUC domains showing distinct patterns among the variants. Notably, the REC domain exhibited high fluctuation between 400 and 450 ns in wildtype AsCas12a. However, the PAM-interacting domains showed fluctuations around 700–750 ns in the RVRm variant. The high fluctuations in certain domains indicates the instability. The average RMSD given in Table 1 states that the different domains of RRm and RVRm attained enhanced stability after mutation. It indicates that the incorporation of mutation in these variants maintains intended conformation over longer timescale.

**Table 1:** Average RMSD of all the variants of CRISPR-AsCas12a system over the extended time scale of 1 μs.

	Wildtype	RR	RVR	RRm	RVRm
**Complex**	2.30	2.79	2.87	2.46	2.09
**REC**	2.12	1.72	2.06	2.05	1.88
**RuvC**	1.87	2.02	1.58	2.13	1.56
**NUC**	1.88	1.80	1.65	1.61	1.80
**PI**	1.85	1.60	1.42	1.54	1.67
**gRNA-DNA hybrid**	1.63	1.49	1.61	1.41	1.51
**PAM Distal**	1.70	1.58	1.11	1.39	1.45
**PAM Proximal**	1.75	1.72	1.38	1.64	1.63

**Fig. 2:**
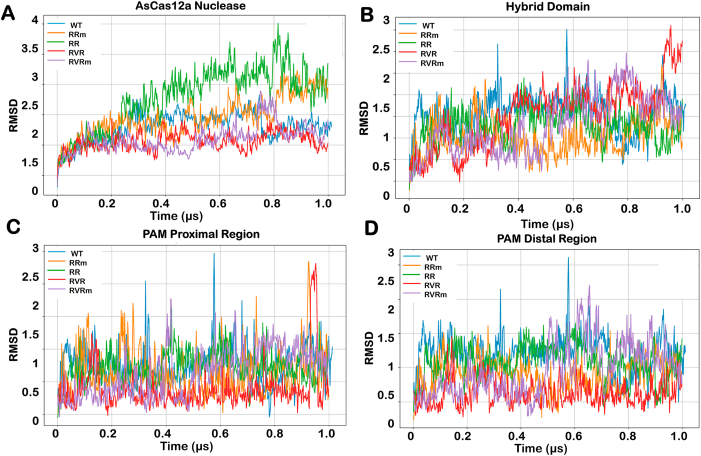
**Stability of different variants of protein complex by root mean square deviation (RMSD) plot.** The comparison of fluctuations over a time frame of 1μs Gaussian accelerated molecular dynamics in (A) AsCas12a, (B) gRNA-DNA hybrid, (C) PAM-pr proximal region and (D) PAM distal region domain. The RMSD profile of gRNA-DNA hybrid indicates the highest fluctuation in hybrid bound to RVR variant at a longer time scale. The PAM-proximal region shows the fluctuation over an extended time frame.

Interestingly, there was a notable instability in PAM proximal region of the gRNA-DNA hybrid towards the end of the simulation in RVR variant. However, after the incorporation of K949A mutation in RVR variant (RVRm), the gRNA-DNA hybrid was stabilized at later time scales indicating the stable binding. The RVR AsCas12a-bound hybrid displayed the most deviation at around 900–950ns in the PAM-proximal region whereas it attained stability after mutation, suggesting the stabilization of complex at non-canonical PAM sites. Fig. 2 represents the stability of five variants of AsCas12a and gRNA-DNA hybrid. Additionally, the structural stability plots of REC, RuvC, NUC and PI domains are given in Supplementary Fig. 1.

### 2.2 Domain-specific flexibility in AsCas12a variants

**Fig. 3:**
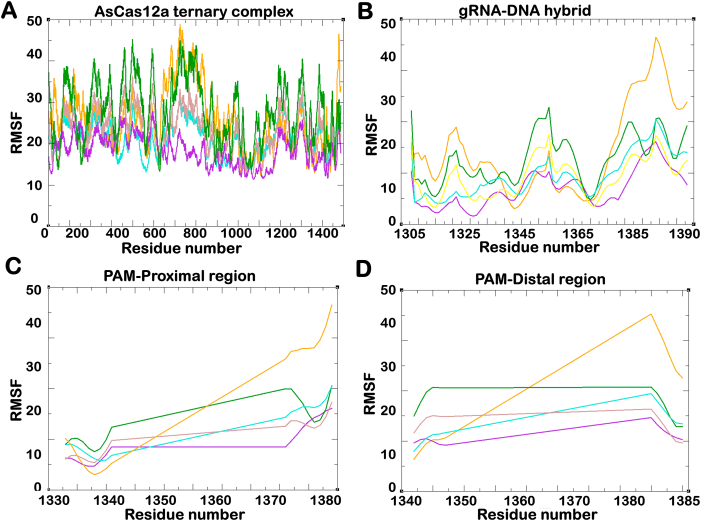
**The residue-wise fluctuation (Ǻ) comparison in Wildtype, RR, RVR, RRm and RVRm variants of AsCas12a.** The root mean square fluctuation (RMSF) of (A) the entire protein, (B) gRNA-DNA hybrid, (C) the PAM proximal region and (D) the PAM distal region represents the flexibility and stability of different domains during the simulation. The position-wise fluctuations in heteroduplexes of different variants indicate the stability of the gRNA-DNA hybrid inside the protein.

The root mean square fluctuation analysis conducted to assess residue-level flexibility revealed higher flexibility in the RVR variant of AsCas12a compared to wildtype AsCas12a across all domains. Specifically, the PAM-interacting domains of both Wildtype and RVR variant AsCas12a exhibited higher fluctuations than the mutants of RR variant and RVR variant. Moreover, Table 2 and Fig. 3 shows that the K949A mutation in RVR variant has induced stability over a longer timescale. The PAM-distal region of Wildtype AsCas12a exhibited maximum flexibility, indicating its ability to tolerate mismatches at canonical PAM site. Fig. 3 shows the fluctuations in the residues of different AsCas12a variant proteins and gRNA-DNA hybrids.

**Table 2:** Average RMSF of all the variants of CRISPR-AsCas12a system over the time scale of 1 μs.

	Wildtype	RR	RVR	RRm	RVRm
Complex	25.41	18.39	27.86	21.30	22.93
REC	24.99	21.34	29.26	21.51	24.93
RuvC	18.92	13.92	24.79	19.15	19.20
NUC	20.88	19.39	28.47	24.43	23.91
PI	39.72	18.29	37.97	26.21	28.20
gRNA-DNA hybrid	24.13	16.96	22.78	19.54	19.15
PAM Distal	28.82	20.89	27.67	23.10	23.29
PAM Proximal	27.38	19.38	23.26	21.95	20.14

The residue level flexibility of REC, RuvC, NUC and PI domains in all the variants is represented in Supplementary Fig. 2. The hybrid showed high flexibility in wildtype and RVR variant complex, whereas stability was attained after K949A mutation in RVR variant. The mutation in AsCas12a PAM variants attained enhance stability closely aligns with the findings of previous study (Hirano et al., 2016; Kleinstiver et al., 2015). These findings provided insights into the structural dynamics and potential mechanistic differences between PAM variants and wildtype AsCas12a. Further analyses were carried to correlate the findings and understand the conformational dynamics.

### 2.3 Coupled motions between the domains of AsCas12a variants

Cross-correlation analysis were further employed to investigate the dynamic conformational changes occurring in the system over a longer time frame. It calculated the correlation between all the atoms in the system and revealed the coupled motions between residues. The aim of this analysis was to capture the overall dependencies of protein domains and explore the interdependent conformational dynamics among spatially distant protein domains. This study highlighted that a few correlated motions were constant between the Wildtype and all the other variants. There was an moderate degree of cross-correlation coefficient between REC domain and Wed II domain during the entire simulation in all variants. Additionally, the RuvC and NUC domains with gRNA-DNA hybrids and a few amino acids of the REC domain with the hybrid were also constant in all the variants. The correlation coefficients between the NUC domain with the REC I and REC II domains were found to be more pronounced in the Wildtype and RVRm than in other variants. However, these coupled motions are present in the wild-type and it exhibits high activity at canonical PAM sites. Therefore, it was used as a reference for comparison with other variants. This analysis revealed an increased correlation coefficient and enhanced fluctuations in REC and NUC domains of RVRm compared to the wild-type. These findings suggest that the K949A mutation in RVR may contribute to improved on-target efficiency by promoting correlated domain motions. The cross-correlation plot for Wildtype, RR and RRm variants of AsCas12a are shown in Supplementary Figure 3-7. In addition to that, WED II shows correlated motions with the PAM Interacting domain and WED III with Bridge Helix in all the variants ensure stable PAM binding. The coupled motion of residues of the NUC domain with the PAM distal region is more prominent in Wildtype AsCas12a than in other variants. The CC_ij_ analysis revealed that the dependent motions between the PAM proximal region and WED III domain are higher in the RVR variant than in Wildtype AsCas12a. RVR variant also exhibited that the residues of the WED III domain exhibited high coupling motions with the REC I and PI domains. Other unique coupled motions concluded from the cross-correlation plot in the RVR variant are between RuvC II with the PI domain and REC II with WED II domains. The top 250 highly positively correlated residues in different variants of AsCas12a are given in Supplementary File 2 for quantitative understanding of the correlation of coupled motions.

**Fig. 4:**
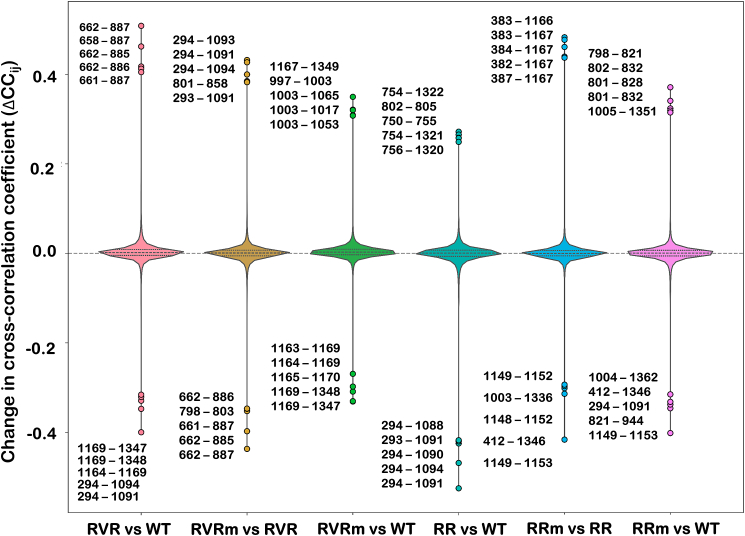
**Distribution of ΔC_ij_ (cross-correlation changes) for residue-residue pairs across different AsCas12a variant combinations.** Each violin represents the distribution of ΔC_ij_ values for a specific comparison. The width of the violin indicates the density of ΔC_ij_ values. Positive ΔC_ij_ values indicate increased correlation, while negative values reflect decreased correlation relative to the compared variant. The five most significant positive and negative outliers for each group are highlighted as colored dots and their corresponding residue pairs are annotated adjacent to the violins. A horizontal dashed line at ΔC_ij_ = 0 serves as the reference for no change**.**

The residue-wise cross-correlation changes (ΔC_ij_) was also calculated to investigate the change in dynamic coupling between residues in different variants of AsCas12a and the distributions are visualized using violin plots as shown in Fig. 4. The comparisons between PAM variants and wildtype AsCa12a showed a broad distribution of ΔC_ij_ values with strong positive and negative outliers indicating widespread incorporation of inter-residue motions. Whereas the PAM variants after K949A mutation showed narrower distributions and fewer extreme outliers when compared to the wildtype, indicating that the K949A mutation further perturbs the internal dynamics of the complexes and might be correlated with improved target efficiency of these variants after K949A mutation. The five most significant positive and negative ΔC_ij_ values for each group, annotated on the plot, highlight specific residue pairs that exhibit the greatest dynamic changes. These residue pairs often involve regions like REC2 and NUC, which are critical for signal propagation and target recognition. The observed distribution of correlated motions may reflect altered allosteric communication pathways that contribute to the variants differential PAM recognition and targeting specificity.

Furthermore, It was observed from the cross-correlation plot that there were no significantly unique correlated motions induced in the RR variant and K949 mutant of the RR variant whereas K949A mutation in the RVR variant induced many new coupled motions compared to Wildtype and other variants. The activation of high intensity coupled motion between REC I and REC II with the NUC domain in RVRm was found more prominent. Coupled motion between the REC2 and NUC domains in wildtype AsCas12a was previously reported by *Saha* et al., and its mechanistic implications were further elucidated in their subsequent study (Saha et al., 2024, Saha et al., 2020). Hence, it was concluded that K949A mutation when associated with K548V, and N552R mutations induced comparatively greater correlated motions. Moreover, It was observed that cross-correlation coefficient between His1167 of the NUC domain and Thr384 of the REC II domain increased during the course of simulation after the K949A mutation. The activation of correlated motion between His1167 and Thr384 in RVRm and RRm is resulting in synchronized motion between REC II and NUC domains. Additionally, Ser959 of the RuvC domain shows high cross-correlation coefficient with multiple residues of the REC II domain in all the variants of AsCas12a. These findings suggest that these interactions may play a role in ensuring high on-target efficiency at non-canonical (TATV) and canonical (TTTV) PAM sites in the mutants of PAM variants.

The additional correlated movements observed between residues of the REC2 and NUC domains after the K949A mutation may reflect enhanced allosteric communication within the complex of PAM variants at both canonical and non-canonical PAM sites. The WED and PI domains are known to be directly involved in PAM recognition and responsible for specificity. In contrary, the coupling motions between residues of REC2–NUC observed in our analysis may contribute indirectly by stabilizing conformational states that facilitate accurate DNA targeting. However, further experimental validation would be required to confirm a link between these domain motions and on-target specificity. There were a few more unique coupled motions between the REC2 domain with BH and the RuvC II domain with the REC II domain were observed in RVRm. Moreover, correlated motion between the WED III domain and PI domain was deactivated in RVRm compared to RVR variant, suggesting a reduction in prominent dynamics responsible for PAM recognition. Additional elevated motions observed in RVRm was between the RuvC II domain with the WED III domain and with the BH domain, as illustrated in Supplementary Figs. 4 and 5. The correlated motions that were prominent across all variants were between PAM-distal region of the gRNA-DNA hybrid with the REC II and NUC domains and the PAM-proximal region with residues of the PI domain. It was found that RVRm and Wildtype AsCas12a has improved domain interaction movements compared to other variants.

The Dynamical Cross-Correlation Matrix analysis was also performed to reveal distinct patterns of correlated motions among the variants. The RR variant exhibited a higher proportion of positively correlated motions compared to negative correlations. The K949A mutation has significantly increased the positive correlation motions in RRm. Similarly, in K949A mutation in RVR variant reduced negatively correlated motions and resulted a shift toward more positive correlations compared to the wildtype. Therefore, it was concluded that the K949A mutation in PAM variants are inducing cooperative movements. These findings also suggest that the mutation influenced global conformational dynamics by promoting coordinated motions within the system. The DCCM plot representing movements between amino acids induced in different variants of AsCas12a during simulation are shown in Supplementary Figures 8-12.

### 2.4 Essential dynamics in the AsCas12a variants and their bound hybrids

Principal component analysis revealed trends in the significant collective conformational changes in Wildtype, variants of AsCas12a, and the associated gRNA-DNA hybrids. Each eigenvector captured variance in the conformational dynamics of the AsCas12a-gRNA-DNA complex. The eigenvector possessing the highest eigenvalue represents the extensive collective motion in the AsCas12a hybrid. In this study, the first three Principal Components - PC1, PC2, and PC3, were projected onto both two-dimensional and three-dimensional spaces to explore the conformational dynamics of the Wildtype AsCas12a, RR variant, RVR variant and mutant of these variants. The PCA plot of the wildtype AsCas12a and other variants indicate the change in the conformational states during simulation. The conformational states of Wildtype AsCas12a, RR variant, RVR variant, RRm, and RVRm at various time intervals were observed during 1μs simulation. Each data point on the PCA plot represents the conformations of the respective variant sampled during the simulation.

The distributions of active conformations among the Wildtype AsCas12a and other variants indicate the domains flexibility during the simulation. The PCA plot showed the transition of conformations is spread during different time scales. The variance of the datapoints at each time interval indicates that wildtype AsCas12a exhibits relatively stable variance up to 800ns followed by increase in variance after 800ns indicating its flexibility at later timescales. In contrast, conformational variance in RR and RRm indicate enhanced stabilization. However, RVR variant shows a significant conformational change over time whereas after K949A mutation in RVR variant induced relative stability. The increase in percentage variance in RVR variants after 1 μs aligns with the finding of cross-correlation analysis indicating the incorporation of new prominent coupled motions in RVRm. Percentage variance of 3 principal components at varied time scale was also estimated. It is defined as the fraction of the total conformational fluctuations captured by each Principal Component during the simulation. Higher percentage variance for PC1 and PC2 are suggestive of dominant motions. The table representing the percentage variance in the conformational dynamics of different variants of protein is given in Table 3.

**Table 3:** Percentage variance in each principal components of proteins in different variants of AsCas12a over an extended time scale of simulation.

Variants	Time (Microsecond)											
	0.0			0.4			0.8			1.0		
	PC1	PC2	PC3	PC1	PC2	PC3	PC1	PC2	PC3	PC1	PC2	PC3
**Wildtype**	55.08	26.61	18.30	48.22	31.26	20.50	61.36	22.41	16.21	57.51	32.11	10.37
**RR**	64.52	20.19	15.27	74.08	14.27	11.64	42.17	31.31	26.51	62.11	25.04	12.83
**RVR**	64.01	22.13	13.85	68.55	21.01	10.42	46.80	34.68	18.51	78.42	16.52	5.04
**RRm**	46.41	28.34	25.24	49.10	33.29	17.60	57.22	32.65	10.12	54.38	34.75	10.86
**RVRm**	46.64	35.47	17.88	47.73	34.33	17.93	50.37	34.31	15.30	81.78	12.86	5.35

**Fig. 5:**
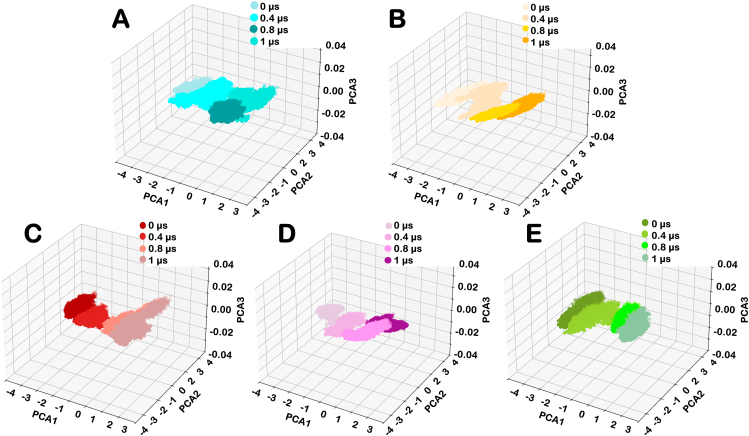
**Principal component analysis (PCA) of five variants of AsCas12a protein, with color variants representing different time frames in each plot.** The three-dimensional representation of principal components over time for (A) Wildtype AsCas12a, (B) RVR variant, (C) Mutant of RVR variant, (D) RR variant, and (E) mutant of RR variant represents the flexibility and conformational dynamics of complexes.

**Fig. 6:**
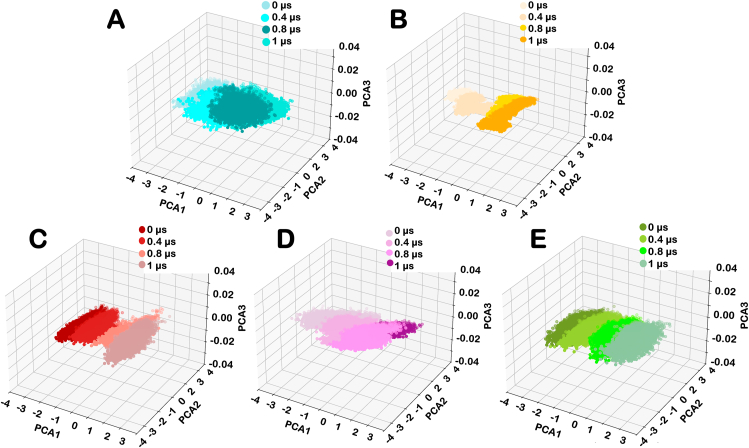
**Principal component analysis (PCA) of gRNA-DNA hybrid bound with five different variants of AsCas12a protein, with color variants representing different time frames in each plot.** The three-dimensional representation of principal components over time for gRNA-DNA hybrid bound with (A) Wildtype AsCas12a, (B) RVR variant, (C) Mutant of RVR variant, (D) RR variant, and (E) mutant of RR variant represents their flexibility and conformational dynamics.

Furthermore, variance among the different conformational ensemble of AsCas12a variants of protein for each principal components during simulation at varied time scale was also estimated and is given in Supplementary Table 1. The PCA plot of RR and RVR variants have spread-out clusters reflecting the diverse range of conformational states present during simulation. It was inferred from the PCA plot that RR and RVR have greater flexibility and conformational spread whereas, after K949A mutation in these variants have stabilized the protein and confined the diversely located data points from the PCA plot. Fig. 5 shows the three-dimensional representation of principal components representing the essential dynamics in wildtype AsCas12a and other variants. The two-dimensional representation of each principal components in all variants of AsCas12a are represented in Supplementary Figures 13-17. The PCA plot of the gRNA-DNA hybrid demonstrated greater flexibility and conformational spread when bound to the RVR variant compared to the other variant. However, the gRNA-DNA hybrid achieved enhanced stability after K949A mutation in RVR variant. The spread of conformational changes over the duration of simulation in three-dimensional space for all variants is shown in Fig. 6. The two-dimensional representation of all the principal components over a varied time frame is shown in Supplementary Figures 18-22.

The average RMSF value given in Table 2 in different domains of RRm is higher compared to the RR variant, whereas the RVR variant exhibits reduced flexibility after mutation, indicating the role of K548V and N552R along with K949A in stabilizing the variants. The PCA plot shows enhanced stability in the Cas12a nuclease in mutants of PAM variants. RMSF primarily reflects residue-level localized flexibility, while PCA provides insights into the overall conformational ensemble generated during the simulation. The observed discrepancy between these analyses arises because RMSF captures local fluctuations of individual residues, whereas PCA identifies global structural motions. Despite the higher RMSF in RRm, the confined clustering observed in PCA plots suggests that the global conformational states remain structurally stable throughout the simulation. This indicates that although certain regions exhibit increased flexibility, these fluctuations do not lead to large-scale destabilization of the protein structure. Furthermore, the stabilization of the RVR variant highlights the synergistic role of K548V and N552R in compensating for the effects of the K949A mutation, ensuring a more stable conformational landscape.

The percentage variance in the conformational dynamics of gRNA-DNA hybrid suggests that the hybrid bound to RVRm exhibits significant conformational changes after 800ns whereas the hybrid bound to other variants are stable throughout the simulation. The table representing the percentage variance in the conformational dynamics of gRNA-DNA hybrid bound to different variants of AsCas12a is given in Table 4. Furthermore, variance among the different conformational ensemble of gRNA-DNA hybrid bound to different variants of AsCas12a for each principal components during simulation at varied time scale was also estimated and is given in Supplementary Table 2. The spread of individual data points highlighted the varying degrees of flexibility and conformational diversity among different variants. The K949A mutation in the RR and RVR variants induced greater stability in the Cas12a-bound hybrid. Overall, PCA elucidated the dynamic conformational landscapes of Wildtype AsCas12a and other variants to provide insights into their structural flexibility and stability.

**Table 4:** Percentage variance in each principal components of gRNA-DNA hybrid in different variants of AsCas12a over an extended time scale of simulation.

Variants	Time (Microsecond)											
	0.0			0.4			0.8			1.0		
	PC1	PC2	PC3	PC1	PC2	PC3	PC1	PC2	PC3	PC1	PC2	PC3
**Wildtype**	48.80	36.27	14.92	56.51	33.32	10.16	60.19	30.90	8.89	65.66	29.99	4.33
**RR**	49.70	38.01	12.27	47.21	32.71	20.07	47.86	35.56	16.57	60.89	29.69	9.40
**RVR**	71.89	22.63	5.47	51.82	34.64	13.53	57.99	30.65	11.35	51.82	37.87	10.30
**RRm**	51.85	33.69	14.45	51.90	34.42	13.66	58.76	26.70	14.53	63.70	24.29	12.00
**RVRm**	51.85	33.69	14.45	51.90	34.42	13.66	58.76	26.70	14.53	63.70	24.29	12.00

### 2.5 Structural flexibility of bases in the hybrid of AsCas12a variants

**Fig. 7:**
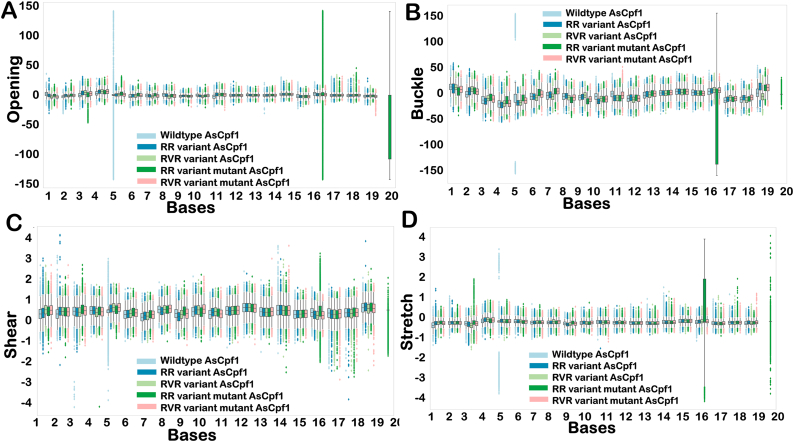
**The representation of structural distortion in the base pair structural parameter “*Shear*”, “*Buckle*”, “*Opening*” and “*Stretch*” in gRNA-DNA hybrid over an extended time frame of 1μs.** Structural base pair fluctuations in the gRNA-DNA hybrid when bound to Wildtype, RR, RVR, mutant of RR, and RVR variant for each position in terms of *Shear*, *Buckle, Opening, Strech* are represented. The generated boxplot has the median in the box and the 25th and 75th quartiles as whiskers in the plot. The data points outside the whiskers are outliers and represent the prominent structural distortions in the respective heteroduplexes.

Base-pair parameters of the gRNA-DNA hybrid were analyzed to understand the structural integrity and stability at the base-pair level (Fig. 7). This analysis depicted that the conformational fluctuations of few bases in gRNA-DNA hybrid varies when bound to different variants of AsCas12a. Most of the estimated structural parameter shows that the position 5th of wildtype AsCas12a and position 16th of RRm variant are significantly more prone to fluctuations. Higher fluctuations in *Buckle, Opening, Stretch at* the position 5th in wildtype AsCas12a from the PAM sequence represent instability and are considered as position more tolerant to mismatches. The position 16th in RRm variant shows high fluctuation in *Buckle, Opening, and Stretch* also indicative of higher flexibility and instability of hybrid during simulation. The structural parameter plot suggests that wildtype AsCas12a can tolerate mismatches at respective positions. However, the K949A mutation in RR variant (RRm) induced higher flexibility at position 16th of gRNA-DNA hybrid*.* It was concluded from this analysis that K949A mutation has induced fluctuations at certain positions in gRNA-DNA hybrid bound to RRm variant. This study suggests that while the K949A mutation induces stability in the Cas nuclease of both PAM variants, the bound hybrid becomes more flexible in the RR variant after the mutation. In contrast, stability of hybrid was retained in the RVR variant even after the mutation. The fluctuations in other structural parameters such as *Stagger* and *Propeller* is shown in Supplementary Figs. 23 and 24.

The fluctuations in structural parameter *shear* across the heteroduplex revealed higher flexibility towards the PAM-distal end in all the variants. It was observed that the hybrid bound to wild-type AsCas12a exhibited more prominent structural deviation in the PAM-proximal region compared to other variants. The structural parameters of gRNA-DNA hybrid bound to RVR and RVRm variant were comparatively stable at all the positions. Most of the positions in all the variants attained a relatively stable conformation. Overall, the base-pair parameter calculations provided insights into the structural dynamics and instability of bound hybrid with the respective variants. The structural parameters “*Shear*” of the gRNA-DNA hybrid over an extended time frame of 1μs is represented in Fig. 7. These findings are consistent with the observed variations in RMSD, RMSF, and conformational entropy values of the gRNA-DNA hybrid.

### 2.6 Conformational flexibility in AsCas12a variants

Conformational entropies of the gRNA-DNA hybrid were calculated to provide insights into the conformational flexibility of the variants by measuring the number of conformations the hybrids can adopt over a timeframe. The conformational entropies of the Wildtype, RR variant, RVR variant, RRm, and RVRm variants were 4053.74 cal/mol-kelvin, 3909.58 cal/mol-kelvin, 4036.35 Cal/mol-kelvin, 3899.61 cal/mol-kelvin, and 3976.58 cal/mol-kelvin, respectively. The conformational entropies of the variants suggest that the gRNA-DNA hybrid is flexible and dynamic when bound to Wildtype and RVR variants. At the same time, the K949A mutation induces more stability in the hybrid of the RR and RVR variants. Additionally, it was concluded that Wildtype AsCas12a has the highest conformational entropy, indicating more accessible conformations over a timeframe.

### 2.7 Analysis of intermolecular polar interactions in AsCas12a variants

Hydrogen bonds are crucial in understanding the stability between gRNA-DNA hybrids and Cas12a nuclease. Therefore, the number of hydrogen bonds between the AsCas12a variants and the bound gRNA-DNA hybrid was estimated to understand the intermolecular polar interactions. The average number of hydrogen bonds between the AsCas12a variants and the gRNA-DNA hybrid is 650 ± 32.34 for wtCas12a, 654.63 ± 33.91 for RVR, 696.00 ± 34.53 for RVRm, 694.88 ± 34.51 for RR, and 705.16 ± 35.13 for RRm. The hydrogen bond analysis revealed that the RRm variant has the highest number of hydrogen bonds within the AsCas12a-gRNA-DNA complex. The statistics of the number of hydrogen bonds also indicate that the K949A mutation has significantly increased the polar interaction between the gRNA-DNA hybrid and AsCas12a variants. The higher number of polar interactions between gRNA-DNA hybrid and AsCas12a variants also shed light on their contribution to attaining stability with gRNA-DNA. The trends shown in RMSD, RMSF, and structural parameters analysis validate this finding.

Furthermore, the hydrogen bonds formed between the protein and gRNA-DNA hybrid during simulation revealed the importance of K949A mutation in attaining stability in variants. The intermolecular hydrogen bond analysis suggests a gain of hydrogen bonds between the WED domain and hybrid of variants as compared to wildtype AsCas12a. Table 5 shows domain-specific hydrogen bond occupancies in Wildtype, RR, RVR, RRm and RVRm variants. The increase in polar interactions between gRNA-DNA hybrid and AsCas12a variants in the WED domain indicates the improvement in DNA recognition and binding mechanism of target DNA with Cas12a. The REC and NUC domains of AsCas12a exhibited similar findings despite some residues in RVR and RRm variants losing polar interactions over the 1μs time frame. Moreover, RVRm variant retained most of the polar interactions present in Wildtype AsCas12a. Table 6, Table 7 illustrate that these variants establish new polar interactions in addition to the existing ones in Wildtype AsCas12a.

**Table 5:** Comparison of domain-wise occupancy of hydrogen bonds retained in Wildtype, RR variant, RVR variant, and mutant of RR and RVR variant of AsCas12a.

Domain	Acceptor	Donor	W	RR	RVR	RRm	RVRm
**WED domain**	U 1312	ARG 19	0.83	–	0.58	0.38	0.20
	G 1370	ARG 543	0.94	0.85	–	0.94	–
	G 1384	TYR 576	0.89	0.96	0.89	0.89	0.86
	DA 1370	ARG 553	–	–	0.13	–	0.96
	A 1324	GLY 757	0.84	0.86	0.61	0.90	0.81
	C 1314	ARG 864	0.84	0.9	0.92	0.86	0.84
	U 1311	ARG 863	–	0.78	0.87	0.88	0.86
	U 1323	HIS 756	–	0.83	0.83	0.89	0.88
	U 1312	LYS 749	0.73	0.80	0.80	0.89	0.82
	DT 1369	ASN 783	0.80	0.16	0.44	0.57	0.18
	C 1314	RG 864	0.85	0.9	0.92	0.87	0.84
**REC Domain**	DG 1354	ASN 279	0.89	0.88	0.88	0.87	0.88
	C 1347	ARG 412	0.80	0.91	0.15	–	0.95
	A 1348	GLN 411	0.80	0.83	–	–	0.83
	A 1335	LYS 308	0.91	0.86	0.86	0.91	0.90
**RuvC domain**	DT 1361	ILE 965	0.99	0.92	0.91	0.97	0.93
	DT 1364	SER 1052	0.31	0.79	0.71	0.35	0.80

Note: W, RR, RVR, RRm, RVRm is the abbreviation of Wildtype, RR variant, RVR variant, mutant of RR and RVR variant of AsCas12a respectively. These PAM variants targets different PAM sites (TTTN, TCTV, TATV).

**Table 6:** Loss in the occupancy of hydrogen bond over the time frame in different variants of AsCas12a.

Acceptor	Donor	W	RR	RVR	RRm	RVRm
DT_1361	ILE_965	0.98	0.9108	0.9208	0.9657	0.9337
DT_1385	THR_168	0.9839	0.9918	0.9881	0.9877	0.9843
DC_1384	TYR_576	0.971	0.9654	0.8946	0.8912	0.862
DT_1385	THR_167	0.9363	0.9457	0.9417	0.9654	0.9468
DA_1370	TYR_596	0.9695	–	–	–	–
DA_1370	TRP_545	0.9339	–	–	0.4755	–
A_1332	TYR_48	0.9227	0.7933	0.8254	0.8769	0.8856
A_1335	LYS_308	0.9062	0.8609	0.8667	0.9119	0.9023
A3_1348	ARG_1169	0.8981	–	0.9414	–	0.1273
U_1333	ARG_177	0.8954	0.6756	0.8073	0.8202	0.8764
DG_1354	ASN_279	0.8866	0.881	0.88	0.8704	0.8781
A_1324	GLY_757	0.8539	–	–	0.9006	0.8072
C_1314	ARG_864	0.8534	0.4252	0.5955	0.8688	0.8419

Note: W, RR, RVR, RRm, RVRm is the abbreviation of Wildtype, RR variant, RVR variant, mutant of RR and RVR variant of AsCas12a respectively. These PAM variants targets different PAM sites (TTTN, TCTV, TATV).

**Table 7:** Loss of hydrogen bond in Wildtype when compared to RVR variant and mutant of RVR variant.

Acceptor	Donor	W	RR	RVR	RRm	RVRm
DT_1361	ARG_1004	–	–	0.93	0.31	0.18
U_1311	ARG_864	0.65	0.9	0.92	0.87	0.84
DG_1356	THR_316	–	0.89	0.89	0.88	0.73
DC_1384	TYR_576	–	0.96	0.89	0.89	0.86
ASN_176	A_1331	–	0.94	0.88	0.48	0.44
U_1311	ARG_863	–	0.78	0.87	0.88	0.86
U_1334	ARG_193	0.69	0.84	0.85	0.75	0.79
U_1323	HIE_756	–	0.83	0.83	0.89	0.88
DA_1353	HIE_301	0.56	0.84	0.80	0.71	0.81

Note: W, RR, RVR, RRm, RVRm is the abbreviation of Wildtype, RR variant, RVR variant, mutant of RR and RVR variant of AsCas12a respectively. These PAM variants targets different PAM sites (TTTN, TCTV, TATV).

There was a gain in polar interactions in the RR variant compared to the Wildtype variant. The K949A mutation increased the stability in both the PAM variant due to increase in hydrogen bond occupancy. These changes in hydrogen bond interactions may contribute to the stability of the PAM-distal region of gRNA-DNA complexed with the mutant of these variants. The gain in hydrogen bonds in a mutant of the RVR variant in the REC and RuvC domains may indicate the increase in activity of these domains, as shown in Table 7. Two unique high hydrogen bonds were formed during simulation in the RVRm as shown in Table 8. In summary, alterations in hydrogen bond interactions between AsCas12a variants and the gRNA-DNA hybrid may influence the stability and dynamics of the complex, potentially impacting the efficiency and specificity of the CRISPR-Cas12a system. Further research is needed to elucidate the mechanistic details of these interactions and their implications for genome editing applications.

**Table 8:** Unique high occupancy hydrogen bond in RVRm variant when compared to Wildtype and other variants.

DNA/RNA	Residue	W	RR	RVR	RRm	RVRm
DA 1370	ARG 553	–	–	–	–	0.96
C 1347	ARG 412	0.79	0.90	–	–	0.95

Note: W, RR, RVR, RRm, RVRm is the abbreviation of Wildtype, RR variant, RVR variant, mutant of RR and RVR variant of AsCas12a respectively. These PAM variants targets different PAM sites (TTTN, TCTV, TATV).

## 3 Methods

### 3.1 Collection of PDB structures of AsCas12a variants

In this study, the PDB structures of Wildtype AsCas12a (5B43), RR variant of AsCas12a (5XH7), and RVR variant of AsCas12a (5XH6) were retrieved from the RCSB-PDB database (Yamano et al., 2016; Nishimasu et al., 2017; Burley et al., 2017). These structures are reported to be resolved by X-ray diffraction technique at resolutions of 2.8 Å for 5B43 and 2 Å for 5XH7 and 5XH6. The crystal structures of AsCas12a bound in a ternary complex with crRNA, target DNA and NTS provide insight into the conformational rearrangements occurring in Cas12a upon binding to target DNA. This study focuses on five variants of AsCas12a. These include the (a) Wildtype, (b) variants with specific mutations at the S542R/K607R positions referred to as RR, (c) variants with mutations at the S542R/K548 V/N552R positions referred to as RVR, (d) RRm and (e) RVRm variants. Mutations present in all variants are summarised in Supplementary Table 3. A schematic representation illustrating the constant crRNA, target DNA strand, and non-target DNA strand among all the variants of AsCas12a and different PAM sites tolerated by respective variants as depicted in Fig. 1(A–D). A recently published study indicated that the K949A mutation in RR (RRm) and RVR (RVRm) variants reduced cleavage at all assessed off-target sites while maintaining high levels of on-target activity (Gao et al., 2017). These variants have different PAM preferences and were selected to understand the role of these mutations in the conformational dynamics of the AsCas12a variant complexes. Fig. 1 (C) shows the mutations within the WED, PAM-interacting (PI), and bridge helix regions of the RR, RVR variants, and mutant forms of these variants. The guide RNA spans residues 1308–1350 and target DNA spans residues 1351–1388 in PDB structure. These spanning paired residues were considered as gRNA-DNA hybrid. The nucleotides closest to the PAM site in target DNA (positions 1–10) were considered as PAM-proximal region, and nucleotides further from the PAM site in target DNA (positions 11–21) were PAM distal region. The range of residues belongs to the respective domains are given in Table 9. The missing residues in the PDB structures 5B43, 5XH6, and 5XH7 were modelled using the Prime package of the Maestro utility from the Schrödinger suite to ensure high-quality loop refinement and side-chain optimization (Schrödinger, 2008). The modelling was carefully conducted and was minimized well during protein preparation. The mutant of RR and RVR variants were constructed by introducing the K949A mutation in the 5XH6 and 5XH7 PDB structures using Pymol (DeLano, 2002). PyMol was chosen to ensure an accurate representation of the mutated residues in the protein structures. The molecular dynamics study of these variants was expected to provide insights into the conformational dynamics in different variants of the CRISPR-Cas12a system.

**Table 9:** Residue-wise domain organization of CRISPR-Cas12a system.

Domain name	Residues range
REC1	24–320
REC2	321–525
RuvC-I	884–940
RuvC-II	957–1066
RuvC-III	1262–1307
NUC	1067–1261
PI	598–791
WED	527–597
Guide RNA	1308–1350
Target DNA	1351–1388
Non target strand	1389–1398

### 3.2 Gaussian-accelerated molecular dynamics simulation

All the PDB structures were pre-processed by removing solvent molecules and assigning protonation states. The system was created around these PDB structures using the Amber ff14SB force field for proteins, which incorporates the ff99bsc1 corrections for DNA and the ff99bsc0 + ꭓOL3 corrections for RNA (Salomon-Ferrer et al., 2013; Maier et al., 2015; Galindo-Murillo et al., 2016; Bana et al., 2010; Zgarbov et al., 2011; Love et al., 2023). All five PDB structures were solvated using the TIP3P water model (Jorgensen et al., 1983). To ensure proper coordination of TIP3P water with ions, Joung/Cheatham ion parameters for TIP3P water were employed (Jorgensen et al., 1983; Joung et al., 2008). These parameters were chosen for their ability to provide a more consistent set of parameters, fitting solvation-free energies, radial distribution functions, ion-water interaction energies, crystal lattice energies, and lattice constants for non-polarizable spherical ions (Joung et al., 2008).

Conventional molecular dynamics (MD) simulations were performed for 100ns to equilibrate the systems and provide starting points for GaMD simulations (Hamelberg et al., 2004, Hamelberg et al., 2007). This process allowed the systems to reach a stable state under the influence of classical mechanics before transitioning to the enhanced sampling technique of Gaussian accelerated molecular dynamics (Hamelberg et al., 2004). In this study, GaMD was preferred over conventional MD due to its ability to enhance conformational sampling and efficiently capture rare events that might not be observed within conventional MD timescales. A time step of 2 fs was utilized, and bond lengths involving hydrogen atoms were constrained using the SHAKE algorithm. Temperature control at 300 K was achieved through Langevin dynamics with a collision frequency of γ = 1/ps. Pressure control was maintained by coupling the system to a Berendsen barostat at a reference pressure of 1 atm and with a relaxation time of 2 ps (Berendsen et al., 1984; Wu and Brooks, 2003). Initially, energy minimization was performed to relax water molecules and counterions, with the protein, RNA, and DNA held fixed using harmonic position restraints of 300 kcal/mol·Å2. Subsequently, the systems were heated from 0 to 100 K in the canonical ensemble (NVT) through two 5ps simulations, with position restraints of 100 kcal/mol·Å2 applied. The temperature was then increased to 200 K over approximately 100 ps of MD runs in the isothermal–isobaric ensemble (NPT), with restraints reduced to 25 kcal/mol·Å2. Following the release of all restraints, the systems were further heated to 300 K in a single NPT simulation lasting 500 ps. After approximately 1.1 ns of equilibration, NPT runs of approximately 10 ns were conducted to stabilize the system density around 1.01 g/cm^3^. Finally, MD simulations of approximately 1 μs were performed in an NVT ensemble for each system. Initially, NPT equilibration was performed to stabilize system density and further production run was conducted in the NVT ensemble to maintain a consistent temperature and ensuring reliable comparative analysis across multiple systems. The GaMD production phase employed a dual-boost potential (igamd = 3), with upper standard deviation limits set to sigma0P = 6.0 for the total potential boost and sigma0D = 6.0 for the dihedral boost. In total, approximately 5 μs of aggregate sampling was collected across five simulation systems (5 systems ∗ ∼1 μs = ∼5 μs). The simulations were conducted using the GPU version of AMBER 18, and the results were analyzed on each simulated MD complex (Salomon-Ferrer et al., 2013).

### 3.3 Principal component analysis

Principal Component Analysis was employed to analyse extensive conformational changes occurring in macromolecules during molecular dynamics simulations (Maisuradze et al., 2009). PCA captures the essential conformational dynamics of the system by reducing the high-dimensional space of atomic coordinates to a lower-dimensional subspace (Yesudhas et al., 2016). In this study, covariance matrix of all the Cα atoms in the protein and DNA-RNA hybrid was calculated and diagonalized to obtain a new set of coordinates called eigenvectors. Each eigenvector has eigenvalues that describe the major conformational dynamics (David and Jacobs, 2014). Therefore, PCA analysis was employed in this study for the five variants of AsCas12a. The complexes were first superimposed to a reference structure to remove rotational and translational motions across compared systems. Further, covariance matrices were constructed from the positions of atoms. Considering, Cij indicate the covariance matrix, where i and j denote atoms i and j. Therefore, Cij was calculated as the average of the displacement vectors for atoms i and j over different time scales. Lastly, the contribution of the total variance was estimated using diagonalized covariance matrix to yield a set of orthogonal collective eigenvectors associated with their respective eigenvalue. The principal component indicating conformational changes induced in different variants of AsCas12a complexes was calculated using the CPPTRAJ module of AmberTools (David and Jacobs, 2014; Stein et al., 2006; Roe et al., 2013).

### 3.4 Cross-correlation analysis

Cross-correlation analysis was conducted to reveal the dynamic coupling of motions between Cα atoms in the AsCas12a-DNA-RNA complexes (Lange and Grubmüller, 2006). A dynamics cross-correlation (CC_ij_) analysis was employed to quantify the collinear correlations between atoms i and j (McCammon, 1984). The CC_ij_ matrix was first computed as normalized covariance matrix between position vectors of atoms i and j over the entire simulation time. Positive CC_ij_ coefficients indicate synchronized motions, negative coefficients suggest anticorrelated motions and CC_ij_ values of zero indicate independence between atoms i and j. The magnitude of CC_ij_ coefficients, ranging from 0 to 1 for synchronized motions and from −1 to 0 for anticorrelated motions, reflects the strength of the correlation. However, it is important to note that the CC_ij_ method neglects nonlinear contributions between atoms i and j and fails to capture correlated motions occurring out of phase with each other.

### 3.5 Analysis of structural stability hybrid bound to AsCas12a variants

The exploration of the conformational space of the gRNA-DNA hybrid during simulations involved assessing structural characteristics related to intra-base and inter-base pair parameters (Babcock et al., 1994; Olson et al., 2001). The nastruct module of cpptraj, a component of AmberTools, was utilized to analyse structural parameters from the trajectory data (Roe et al., 2013). Specifically, *buckle, opening, shear, stretch, stagger,* and *propeller* were examined to comprehend the primary movements associated with base pairing and erosion of the hybrid structure. The goal was to detect discrepancies by comparing the calculated hybrid mechanics' parameters of the Cas12a variants with those of the wildtype AsCas12a1 hybrid.

### 3.6 Conformational entropy

Changes in conformational entropy reflect flexibility alterations, indicating organization and randomness within a biomolecule (Lebrun and Lavery, 1999; Okonogi et al., 2002; Podestà et al., 2005; Williams and Maher, 2000). Thus, conformational entropy was calculated to assess the flexibility of the gRNA-DNA hybrid when bound to Wildtype AsCas12a and other variants. Conformational entropy was computed using a quasi-harmonic oscillation approximation with the cpptraj module of Amber18. Structures were superimposed onto a reference structure to eliminate translational and rotational motions. True conformational entropy (ST) was determined using the conformational probability distribution density, assuming the motion probabilities of 3N atoms follow a multivariate Gaussian distribution to generate an upper bound of true conformational entropy (Hensen et al., 2010; Karplus and Kushick, 1981).

## 4 Conclusion

Off-target activity resulting from mismatches at specific positions hinders the application of the CRISPR/Cas system despite its immense potential for genome editing. The careful design of guide RNAs (gRNAs) while considering nucleotide positional preferences is essential to control off-target activity. Moreover, off-target cleavage can occur due to the activity of Cas nuclease, particularly in cases of imperfect complementarity between the gRNA and off-target sites. The unintended activity of Cas12a emphasizes the critical roles of its recognition and cleavage mechanisms. This study focused on the Wildtype and PAM variants of AsCas12a as the requirement of TTTV PAM in the target sequence limits the utility of Cas12a. Engineered PAM variants of AsCas12a namely, RR (S542R/K607R) and RVR (S542R/K548 V/N552R) variants reported in a study for expanding the target space. This study demonstrates stable root-mean-square deviations across all AsCas12a variants. The RMSF plot showed increased flexibility in most domains of the RVR variants and the wild type, whereas the K949A mutation enhanced stability in both the PAM variants. This observation is consistent with previous findings that suggest comparable indel activity of the RVR variant with the wild type and improved on-target activity at both canonical and non-canonical PAM sites after K949A mutation (Gao et al., 2017; Nishimasu et al., 2017). A notable finding of this study is that the K949A mutation in the RVR variant has induced multiple positive coupled residue movements in the NUC domain with the REC II domains. More importantly, the cross-correlation coefficient between His1167 of the NUC domain and Thr384 of the REC II domain has increased over the entire simulation after the K949A mutation. Additionally, Ser959 of the RuvC domain demonstrates a high cross-correlation coefficient with several residues in the REC II domain across all the variants of AsCas12a. These findings suggest that these interactions may play critical role in binding and recognition target sites at canonical and no-canonical PAM sites in the mutants of PAM variants. His1167, Thr384, and Ser959 are identified and concluded as critical residues involved in the target binding and cleavage activity in all AsCas12a variants. These residues may be further mutated to enhance activity of the variants at both canonical and non-canonical PAM sites. Furthermore, this study observed the presence of greater coupled residue movements in RVRm compared to RRm and concluded the combined influence of K548 V/N552R mutations with K949A is responsible for this difference. This observation indicates that K949A in association with K548V and N552R mutations induces cross-correlated motions. The RMSF analysis indicated pronounced fluctuations in the PAM-proximal region of the RVR variant whereas the overall gRNA-DNA hybrid was stabilized after K949A mutation. The principal component analysis also reveals extensive conformational spread in the RVR variant compared to the clusters obtained from other variants. The principal components in each clusters become more confined after K949A mutation in the PAM variants. PCA of the gRNA-DNA hybrid indicates a higher conformational spread in RVR variants and lower essential dynamics in both variants after mutation. It was concluded that K949A mutation induced enhanced stability in the variants. This study also suggested that engineering His1167, Thr384, and Ser959, in mutants of PAM variants could further improve the specificity and efficiency of the variants for targeting canonical and non-canonical PAM sites. Overall, this study provide insights into the role of K949A mutation in improving stability of PAM variants and predicted critical residues such as His1167, Thr384 and Ser959 for inducing further stability in mutants of PAM variants.

## Authors contribution

P.K.: Conceptualization, Data curation, Formal analysis, Investigation, Methodology, Writing original draft; and D.S.: Conceptualization, Supervision, Writing-reviewing and editing.

## Conflict of interest

No conflict of interest.

## Declaration of competing interest

The authors declare that they have no known competing financial interests or personal relationships that could have appeared to influence the work reported in this paper.

## Data Availability

No data was used for the research described in the article.
