# Supplementary material for: Conformational changes induced by K949A mutation in the CRISPR-Cas12a complex drives an effective target-binding mechanism
Source: Curr Res Struct Biol. 2025 Aug 8;10:100173. doi: 10.1016/j.crstbi.2025.100173 (PMC12392662; doi:10.1016/j.crstbi.2025.100173)
Supplement: Multimedia component 2 [file mmc2.docx]

**Conformational changes induced by K949A mutation in the CRISPR/Cpf1 complex drives an effective target-binding mechanism**

Pragya Kesarwani^1,2^ and Durai Sundar^2,3,4,*^

^1^Regional Centre for Biotechnology, Faridabad 121001, Haryana, India

^2^Department of Biochemical Engineering and Biotechnology, Indian Institute of Technology (IIT) Delhi, New Delhi 110016, India

^3^Yardi School of Artificial Intelligence, Indian Institute of Technology (IIT) Delhi, New Delhi 110016, India

^4^Institute of Bioinformatics and Applied Biotechnology (IBAB), Bengaluru 560100, India

^*^Correspondence: DS: [sundar@dbeb.iitd.ac.in](mailto:sundar@dbeb.iitd.ac.in)

PK: [pragya.kesarwani@rcb.res.in](mailto:pragya.kesarwani@rcb.res.in)

DS: [sundar@dbeb.iitd.ac.in](mailto:sundar@dbeb.iitd.ac.in)

**List of supplementary Tables**

**Supplementary Table 1: Variance in each principal components of proteins in different variants of AsCas12a over an extended time scale of simulation**

| **Variants** |  | **Time (Microsecond)** | | | | | | | | | | | |
| --- | --- | --- | --- | --- | --- | --- | --- | --- | --- | --- | --- | --- | --- |
|  | **Measure** | **0.0** | | | **0.4** | | | **0.8** | | | **1.0** | | |
|  |  | **PC1** | **PC2** | **PC3** | **PC1** | **PC2** | **PC3** | **PC1** | **PC2** | **PC3** | **PC1** | **PC2** | **PC3** |
| **Wildtype** | **Variance** | 0.41 | 0.64 | 0.21 | 0.39 | 0.63 | 0.19 | 0.36 | 0.62 | 0.17 | 0.74 | 1.05 | 0.38 |
| **RR** | **Variance** | 0.46 | 0.39 | 1.01 | 0.31 | 0.92 | 0.96 | 0.19 | 0.81 | 0.93 | 0.21 | 0.34 | 1.05 |
| **RVR** | **Variance** | 0.04 | 0.07 | 0.83 | 0.49 | 0.27 | 0.88 | 0.04 | 0.89 | 1.21 | 0.06 | 2.41 | 1.03 |
| **RRm** | **Variance** | 0.14 | 0.99 | 0.99 | 0.12 | 0.97 | 0.71 | 0.14 | 0.95 | 0.98 | 0.16 | 1.05 | 0.80 |
| **RVRm** | **Variance** | 0.04 | 0.69 | 0.73 | 0.07 | 0.80 | 0.57 | 0.11 | 0.70 | 1.14 | 0.10 | 1.4 | 0.57 |

**Supplementary Table 2: Variance in each principal components of gRNA-DNA hybrid in different variants of AsCas12a over an extended time scale of simulation**

| **Variants** | **Measures** | **Time (Microsecond)** | | | | | | | | | | | |
| --- | --- | --- | --- | --- | --- | --- | --- | --- | --- | --- | --- | --- | --- |
|  |  | **0.0** | | | **0.4** | | | **0.8** | | | **1.0** | | |
|  |  | **PC1** | **PC2** | **PC3** | **PC1** | **PC2** | **PC3** | **PC1** | **PC2** | **PC3** | **PC1** | **PC2** | **PC3** |
| **Wildtype** | **Variance** | 0.11 | 0.26 | 0.28 | 0.23 | 0.84 | 1.00 | 0.08 | 0.28 | 0.20 | 0.16 | 0.71 | 0.36 |
| **RR** | **Variance** | 0.87 | 0.16 | 0.47 | 0.05 | 0.86 | 0.98 | 0.10 | 0.72 | 0.57 | 0.14 | 0.11 | 0.58 |
| **RVR** | **Variance** | 0.06 | 0.79 | 0.61 | 0.24 | 1.48 | 0.61 | 0.06 | 0.33 | 0.30 | 0.03 | 0.72 | 1.54 |
| **RRm** | **Variance** | 0.06 | 1.41 | 1.03 | 0.16 | 0.88 | 0.65 | 0.07 | 0.44 | 1.32 | 0.07 | 0.61 | 0.64 |
| **RVRm** | **Variance** | 0.12 | 0.37 | 0.26 | 0.09 | 0.22 | 0.29 | 0.05 | 0.66 | 0.41 | 0.08 | 2.43 | 0.79 |

**Supplementary Table 3: Summary of all variants used in this study with their respective mutations**

| **Variants** | **Mutations** |
| --- | --- |
| Wildtype AsCpf1 | No mutation |
| RR variant AsCpf1 | S542R, K607R |
| RVR variant AsCpf1 | S542R, K548V, N552R |
| RVRm AsCpf1 | S542R, K548V, N552R, K949A |
| RRm AsCpf1 | S542R, K607R, K949A |

**List of supplementary Figures**


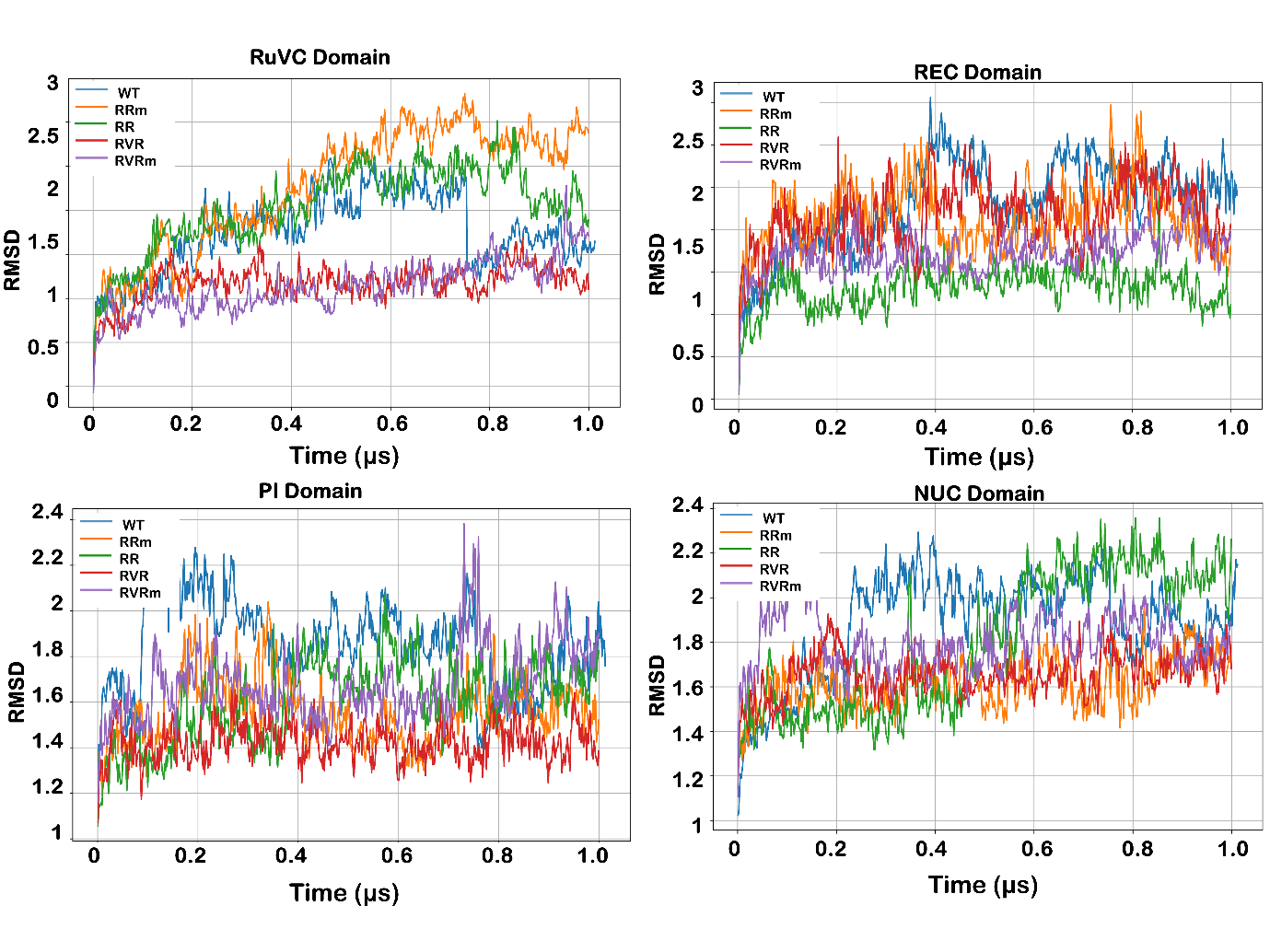


**Supplementary Figure 1: Stability of different variants of protein complex.** The comparison of fluctuations over a time frame of 1µs Gaussian accelerated molecular dynamics in (A) RuVC, (B) REC, (C) Pam interacting and (D) NUC domain.


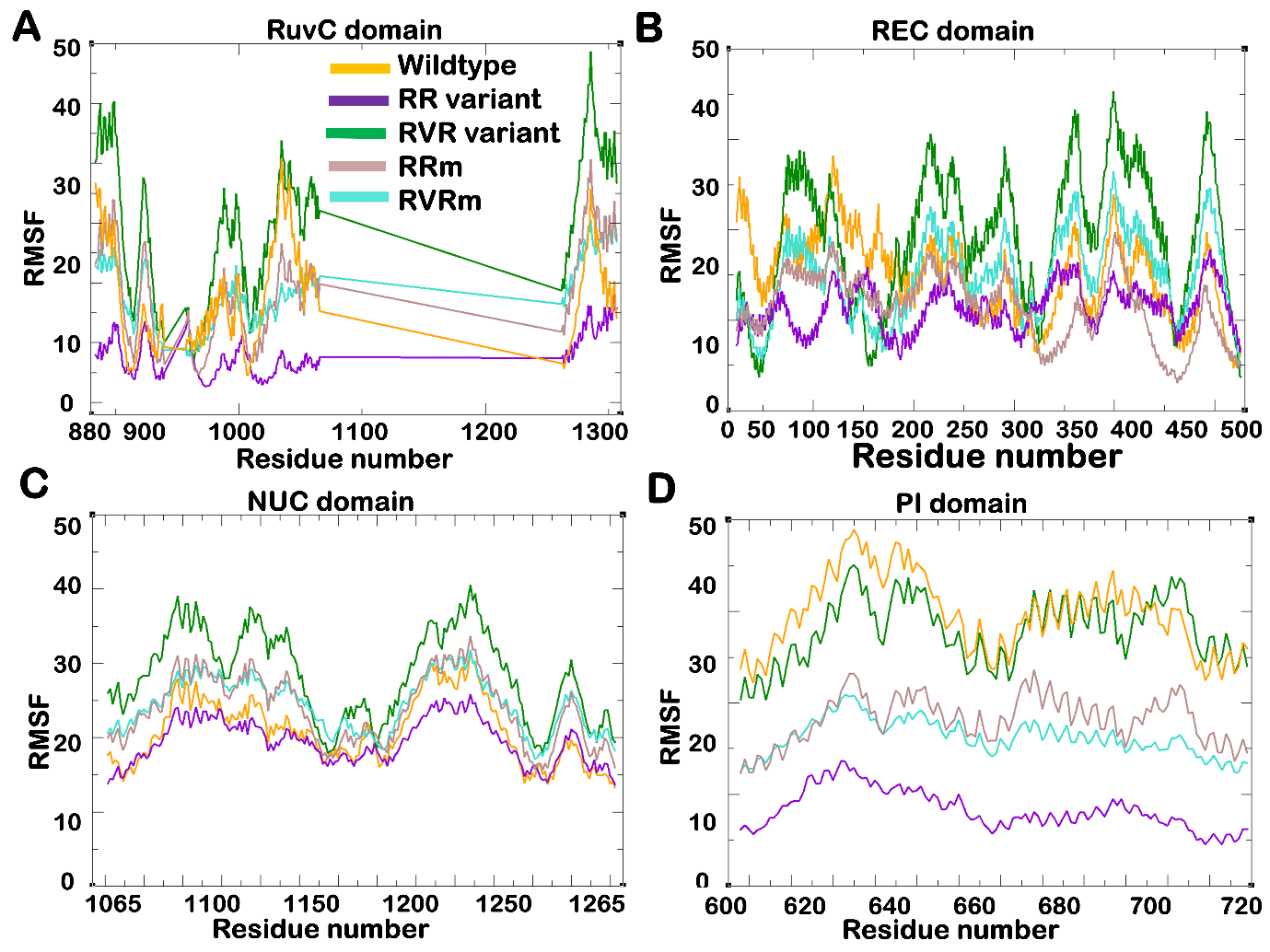


**Supplementary Figure 2:The residue-wise fluctuation (Ǻ) comparison in Wildtype, RR, RVR, K949A mutant of RR and RVR variants of AsCpf1.** The root mean square fluctuation (RMSF) of (A) RuVC, (B) the REC domain, (C) the PI domain, and (E) the NUC domain represents the flexibility and stability of different domains during the simulation.

**
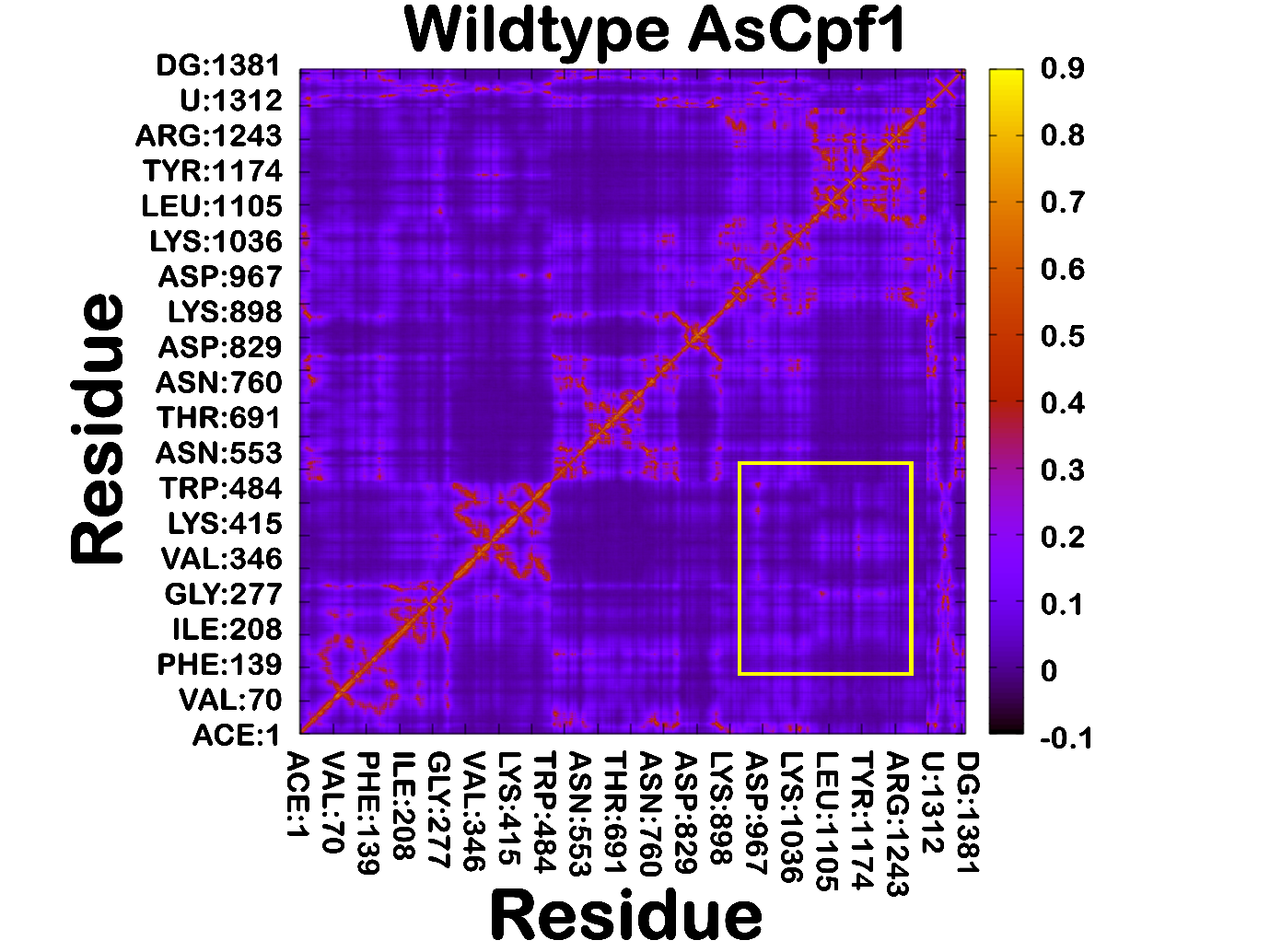
**

**Supplementary Figure 3: Correlated movement of amino acids induced in Wildtype AsCpf1 during simulation**. The colour in the matrix represents the intensity of correlated motion in the AsCpf1-gRNA-DNA complex.


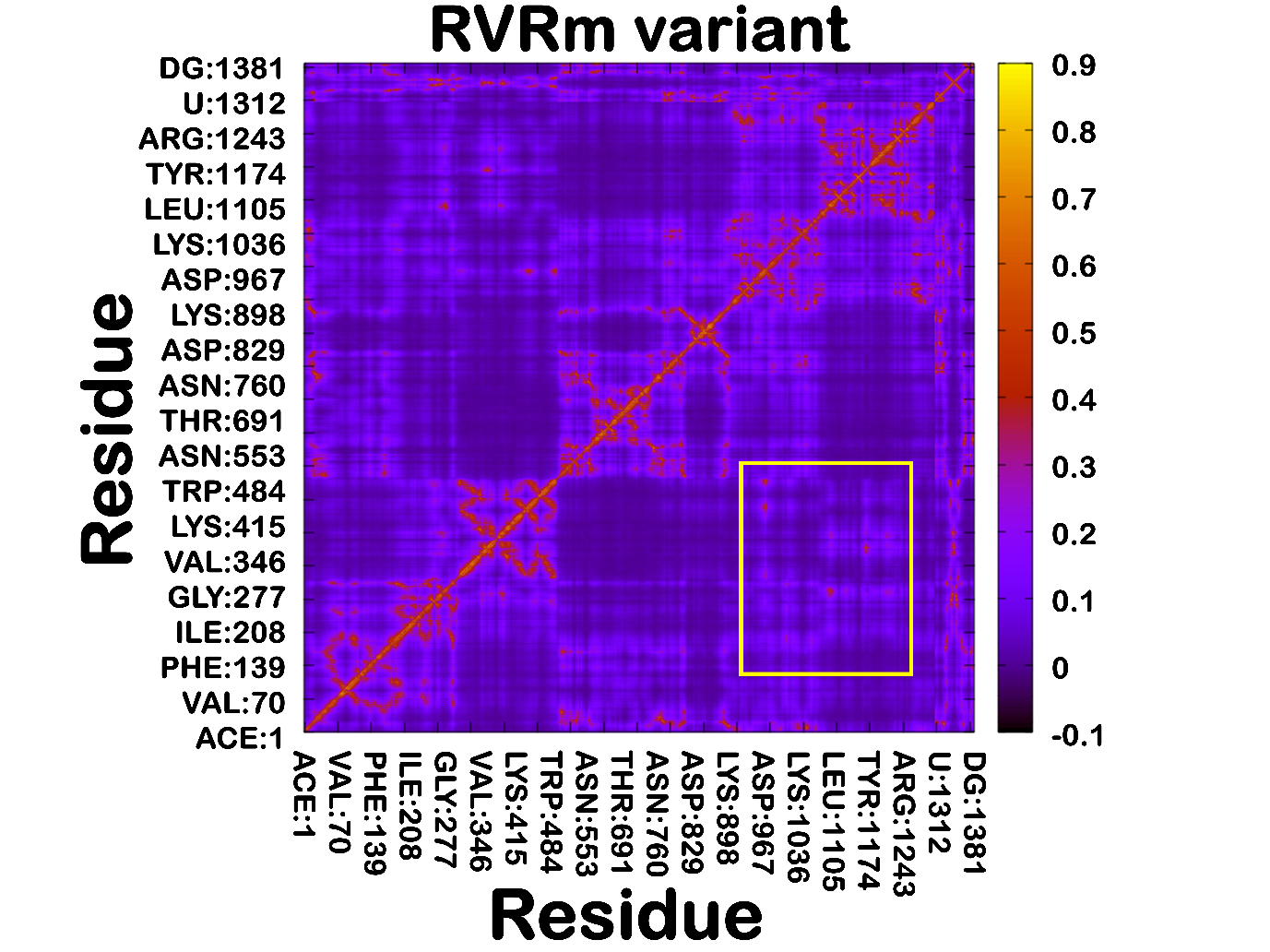


**Supplementary Figure 4: Correlated movement of amino acids induced in RVRm AsCpf1 during simulation. The colour in the matrix represents the intensity of correlated motion in the AsCpf1-gRNA-DNA complex.**


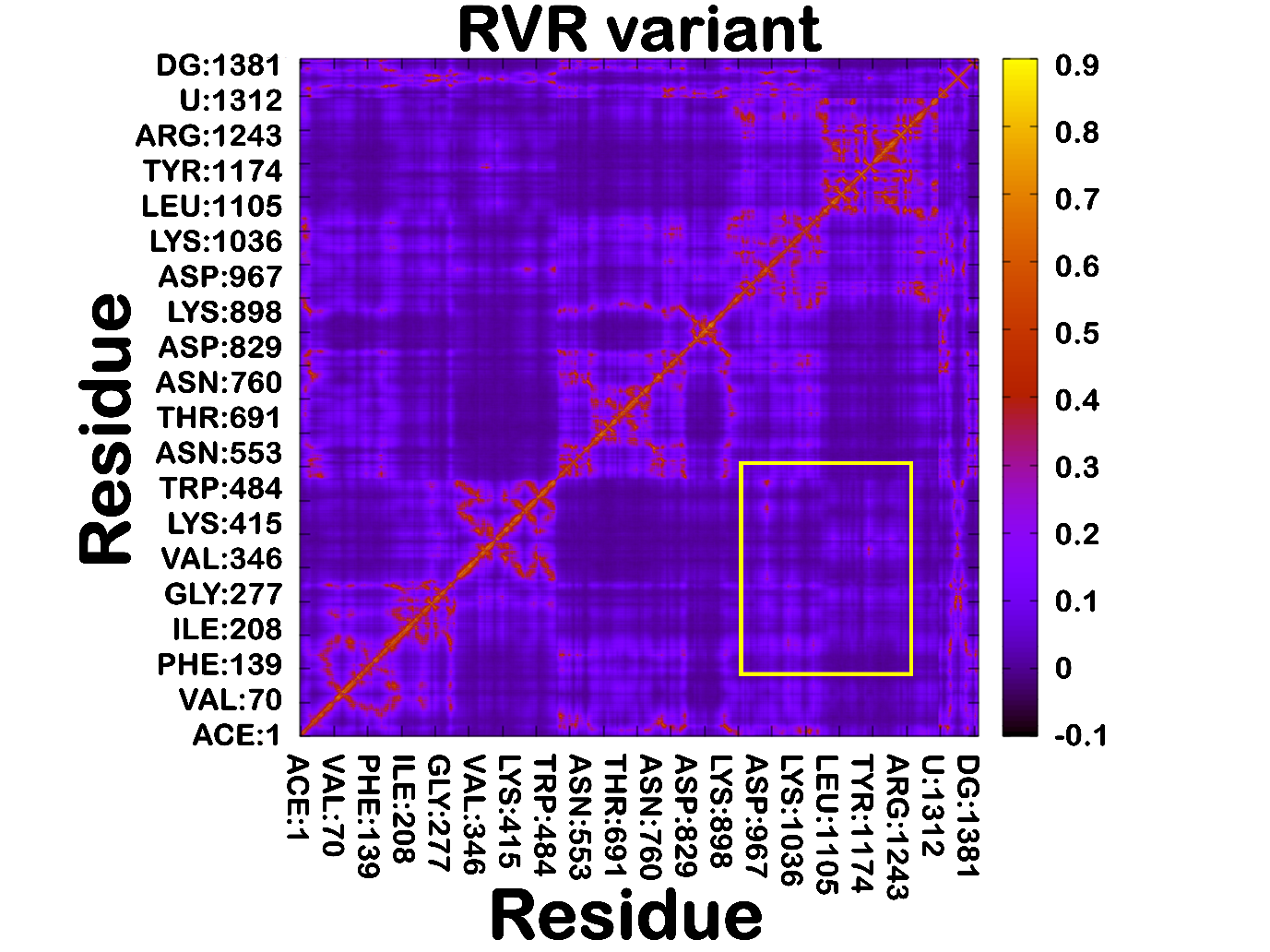


**Supplementary Figure 5: Correlated movement of amino acids induced in RVR AsCpf1 during simulation. The colour in the matrix represents the intensity of correlated motion in the AsCpf1-gRNA-DNA complex.**

**
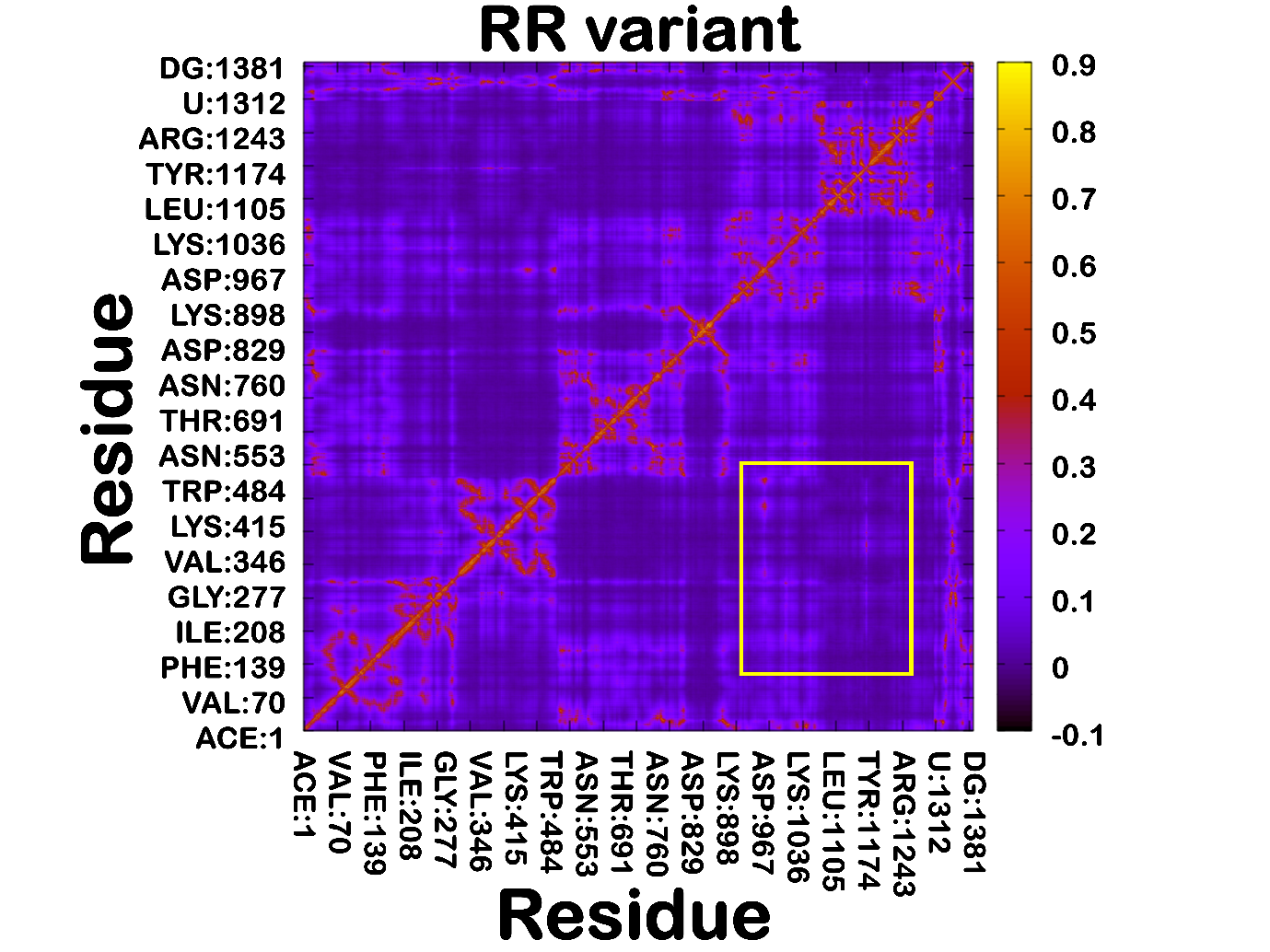
**

**Supplementary Figure 6:Correlated movement of amino acids induced in RR variant of AsCpf1 during simulatio**n. The colour in the matrix represents the intensity of correlated motion in the AsCpf1-gRNA-DNA complex.

**
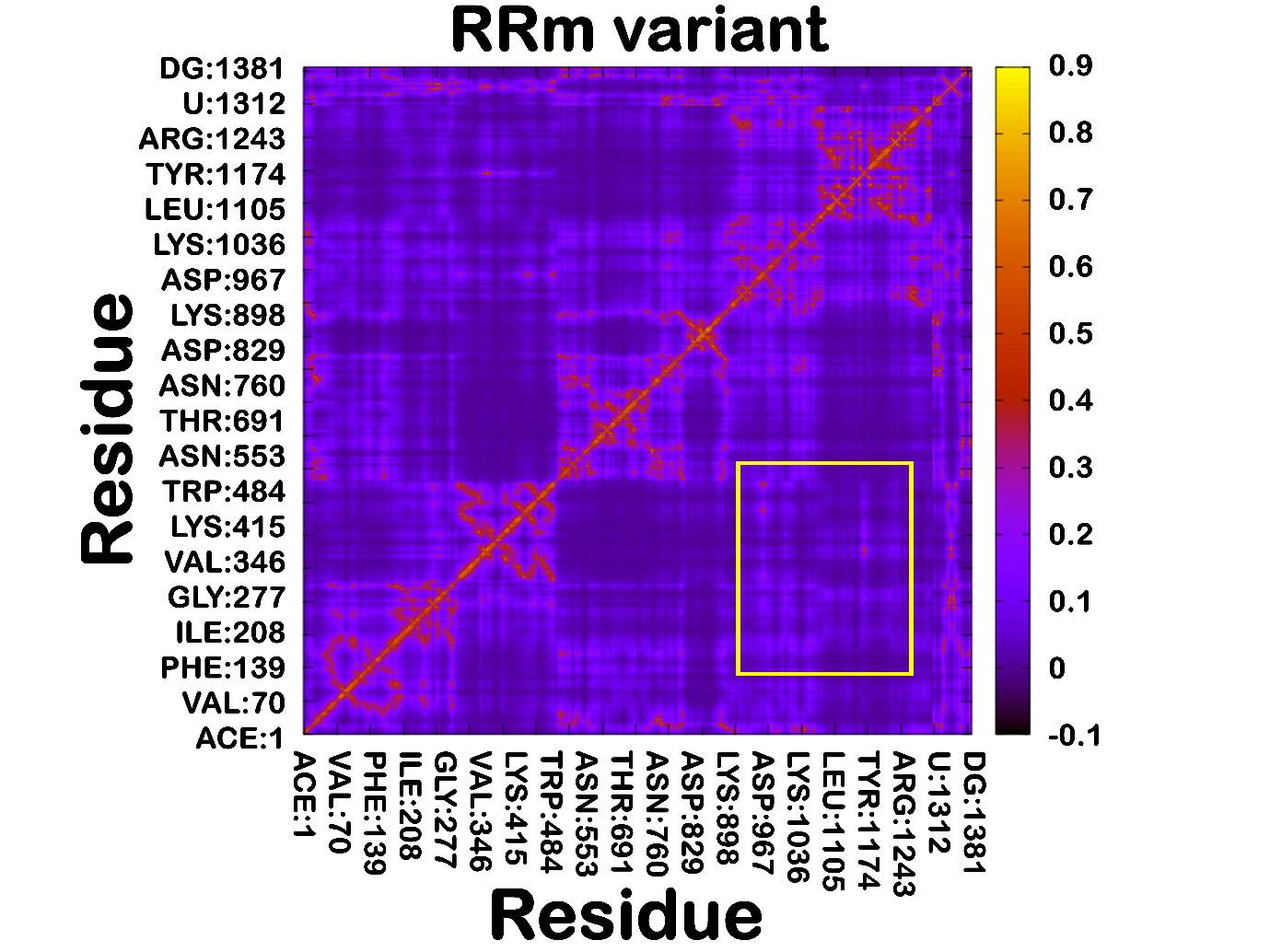
**

**Supplementary Figure 7:Correlated movement of amino acids induced in K949A mutation of RR variant of AsCpf1 during simulation**. The colour in the matrix represents the intensity of correlated motion in the AsCpf1-gRNA-DNA complex.


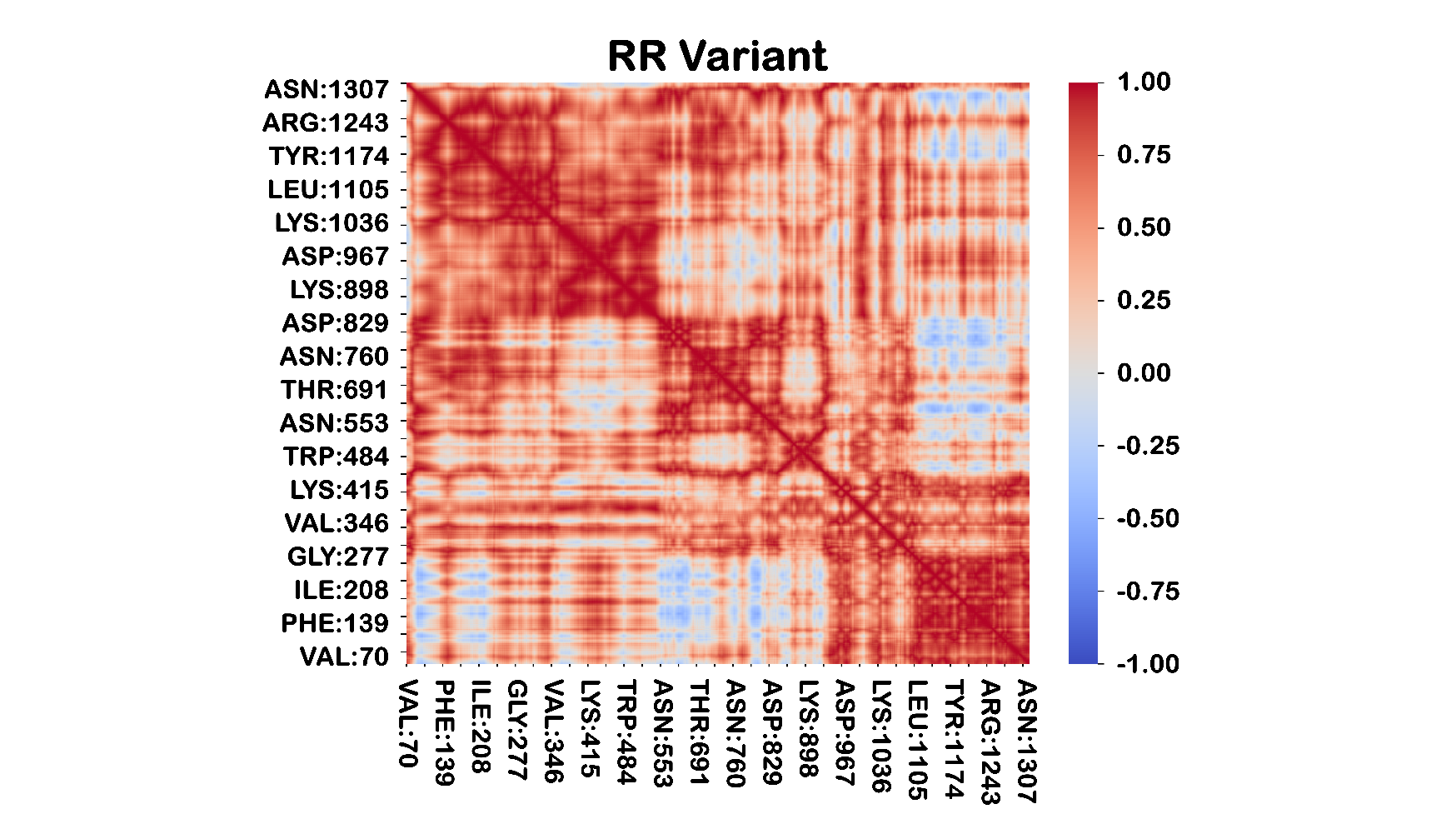


**Supplementary Figure 8: Dynamics cross-correlation movement of amino acids induced in RR variant of AsCas12a during simulation.** The colour in the matrix represents the intensity of correlated motion in the AsCas12a-gRNA-DNA complex.


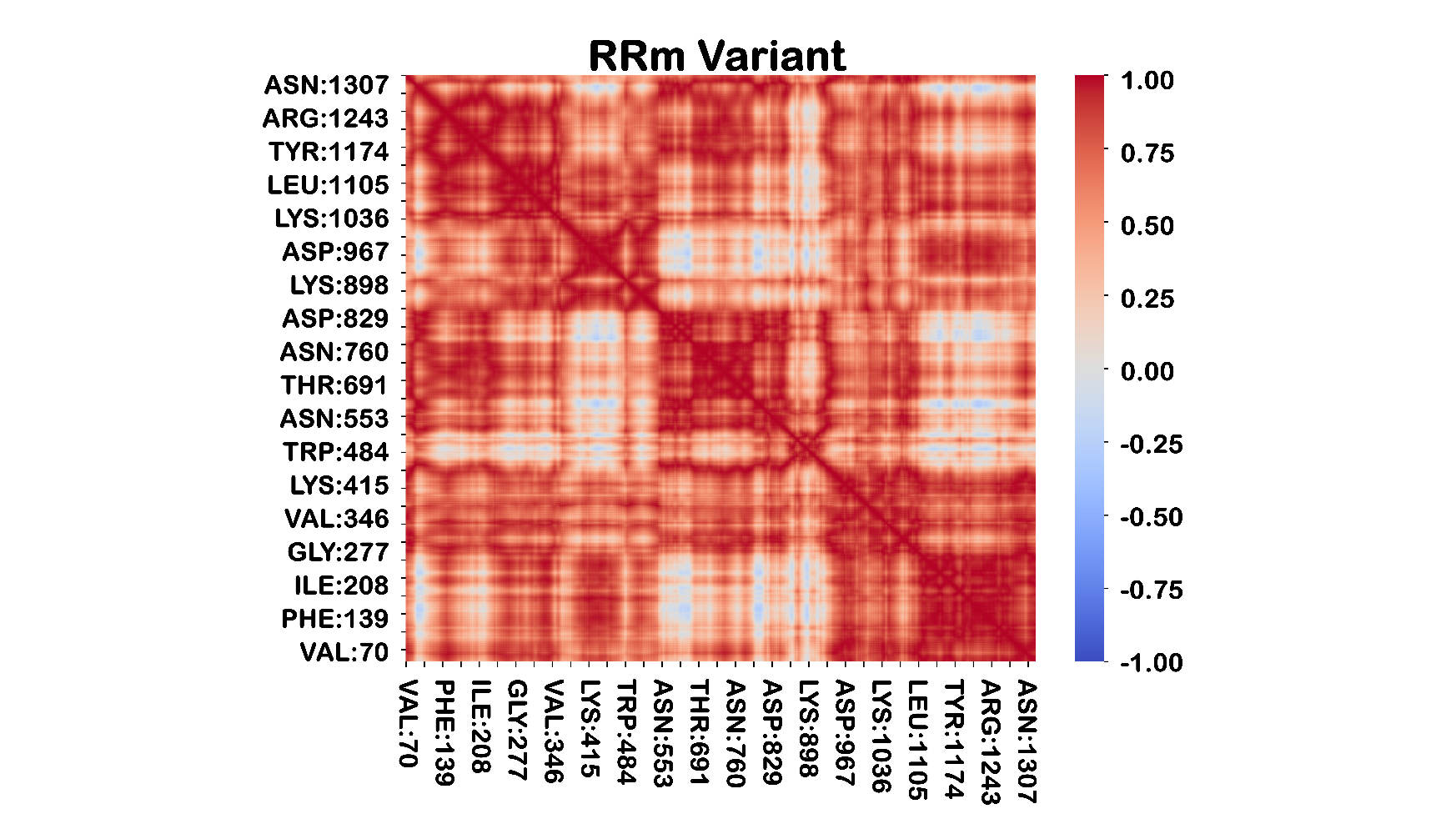


**Supplementary Figure 9: Dynamics cross-correlation movement of amino acids induced in K949A mutation of RR variant of AsCas12a during simulation.** The colour in the matrix represents the positively and negatively correlated motion in the AsCas12a-gRNA-DNA complex.


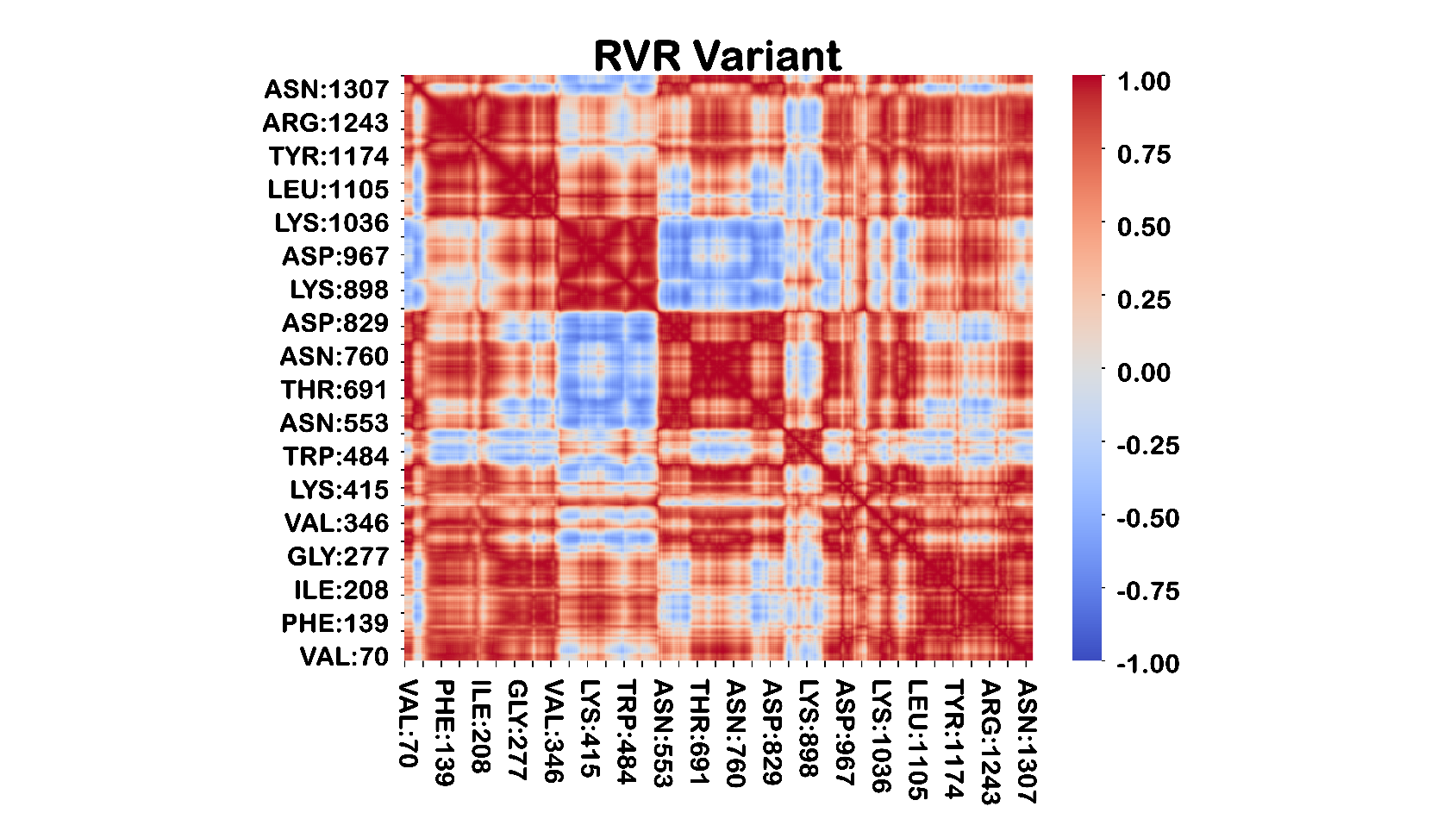


**Supplementary Figure 10: Dynamics cross-correlation movement of amino acids induced in RVR variant of AsCas12a during simulation.** The colour in the matrix represents the positively and negatively correlated motion in the AsCas12a-gRNA-DNA complex.


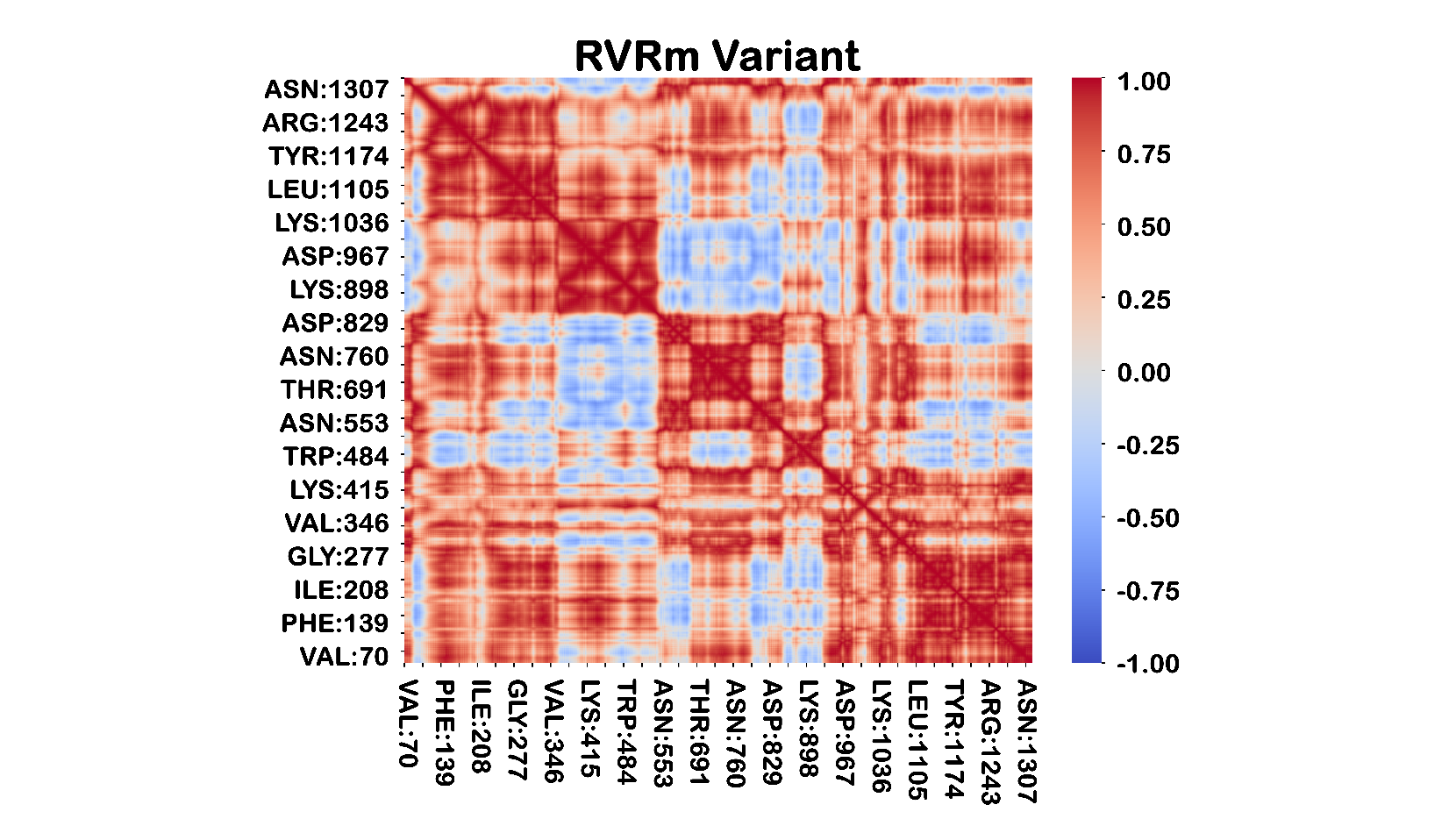


**Supplementary Figure 11: Dynamics cross-correlation movement of amino acids induced in the K949A mutant of RVR variant of AsCas12a during simulation**. The colour in the matrix represents the positively and negatively correlated motion in the AsCas12a-gRNA-DNA complex.


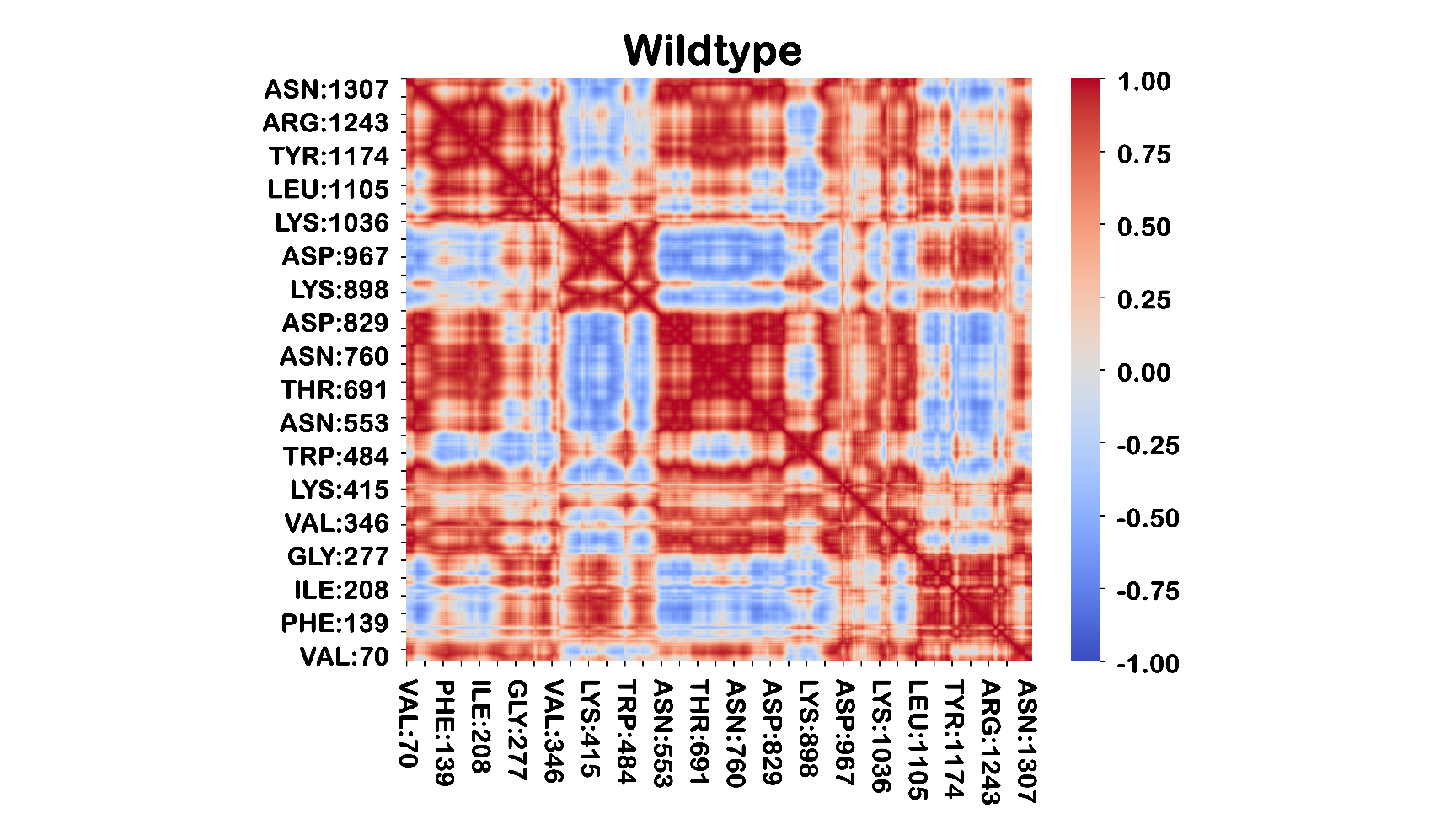


**Supplementary Figure 12: Dynamics cross-correlation movement of amino acids induced in Wildtype AsCas12a during simulation.** The colour in the matrix represents the positively and negatively correlated motion in the AsCas12a-gRNA-DNA complex.


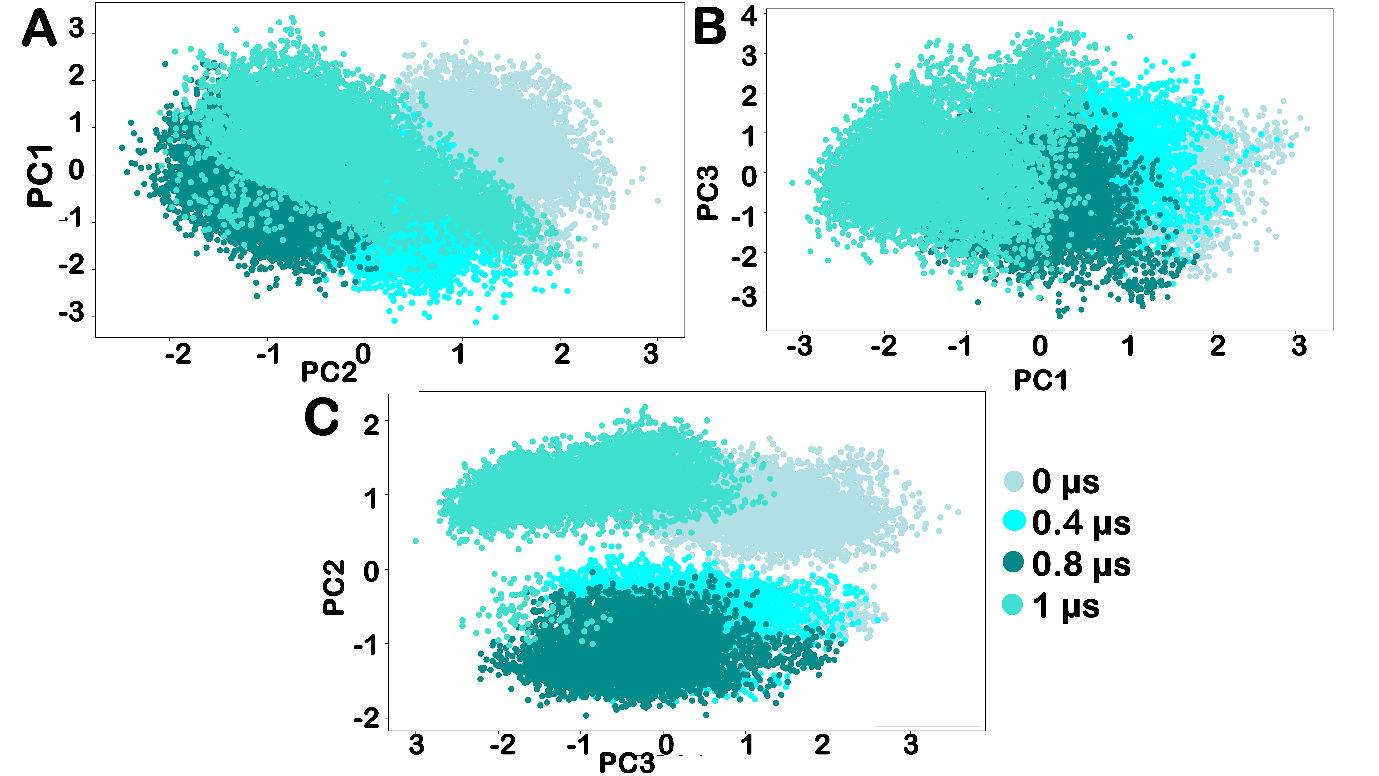


**Supplementary Figure 13:Principal component analysis (PCA) of gRNA-DNA hybrid bound with Wildtype AsCpf1 protein, with colour variants representing different time frames in each plot.** The 2-D plot represents the stability in hybrid bound to Wildtype AsCpf1. All three principal components shown in two dimensions represent the essential conformational dynamics of gRNA-DNA hybrid complexed with Wildtype AsCpf1.


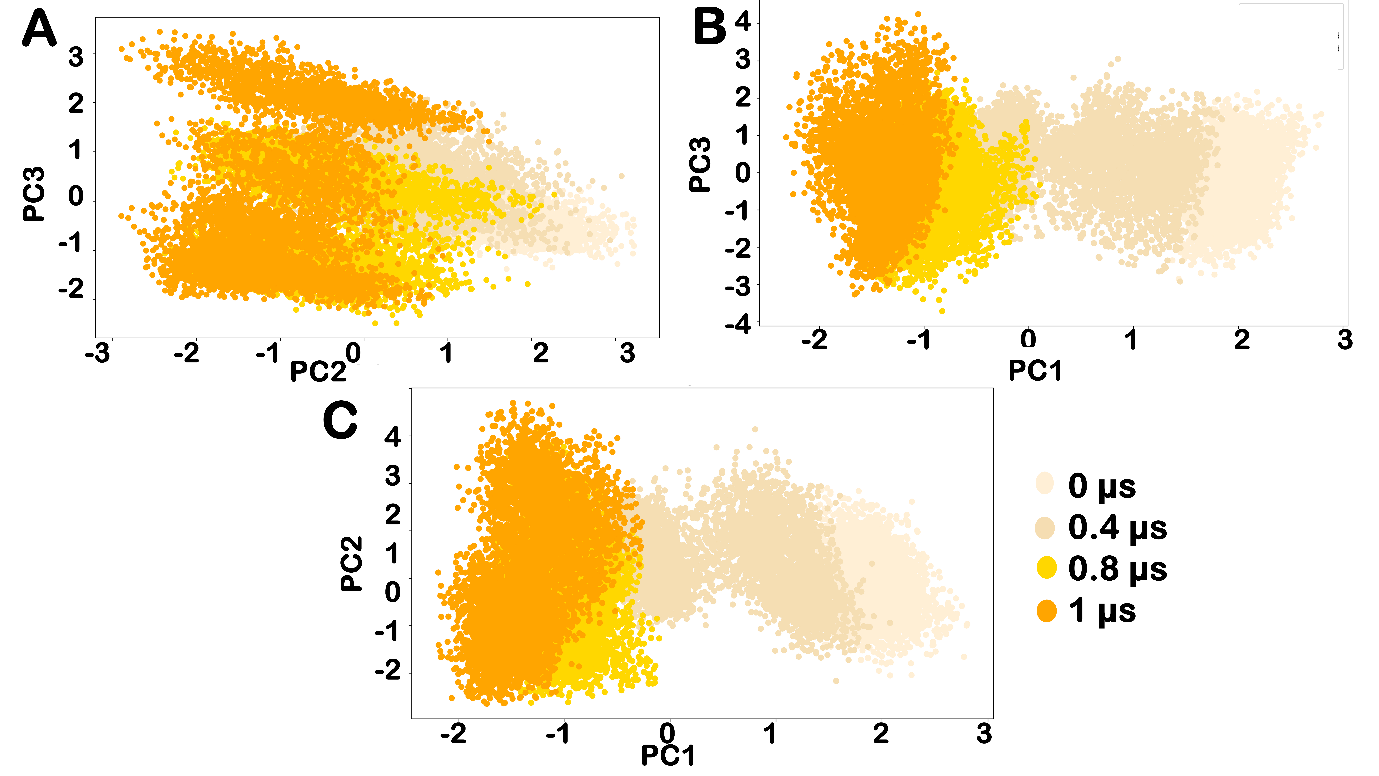


**Supplementary Figure 14: Principal component analysis (PCA) of gRNA-DNA hybrid bound with RVR variant of AsCpf1 protein, with colour variants representing different time frames in each plot.** The 2-D plot represents the stability in hybrid bound to RVR variant. All three principal components shown in two dimensions represent the essential conformational dynamics of gRNA-DNA hybrid complexed with RVR variant of AsCpf1.


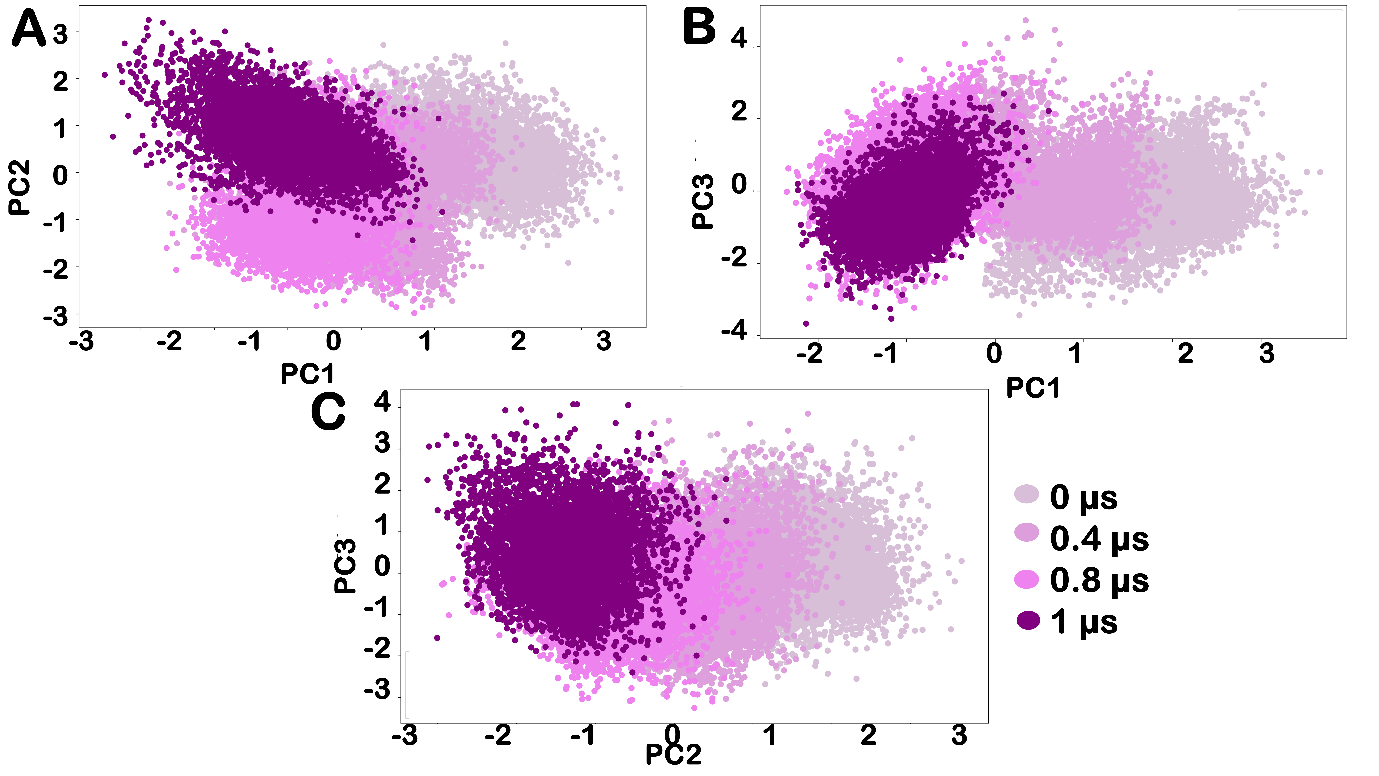


**Supplementary Figure 15: Principal component analysis (PCA) of gRNA-DNA hybrid bound with RR variant of AsCpf1 protein, with colour variants representing different time frames in each plot.** The 2-D plot represents the Stability in hybrid bound to RR variant. All three principal components shown in two dimensions represent the essential conformational dynamics of gRNA-DNA hybrid complexed with RR variant of AsCpf1.


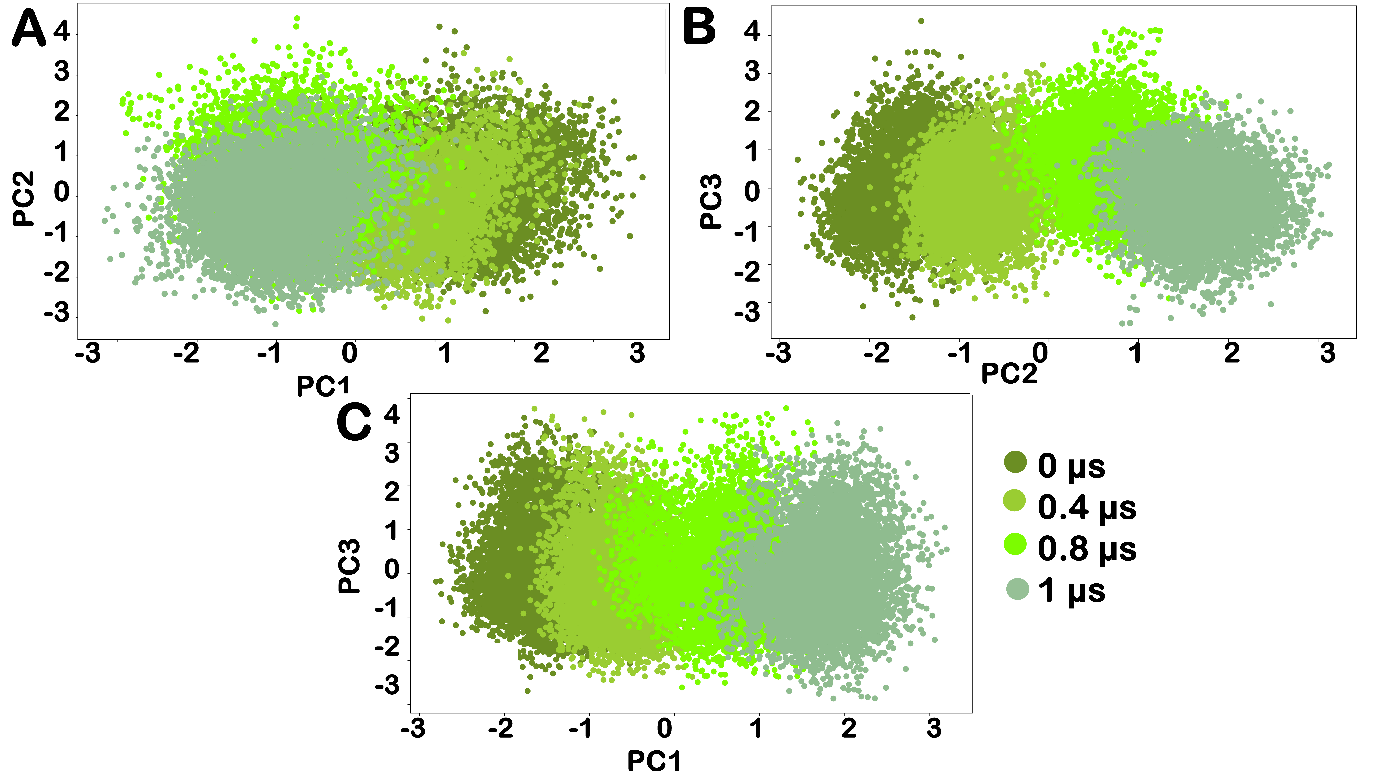


**Supplementary Figure 16: Principal component analysis (PCA) of gRNA-DNA hybrid bound with K949A mutant of RR variant of AsCpf1 protein, with colour variants representing different time frames in each plot.** The 2-D plot represents the Stability in hybrid. All three principal components shown in two dimensions represent the essential conformational dynamics of gRNA-DNA hybrid complexed with K949A mutant of RR variant of AsCpf1.


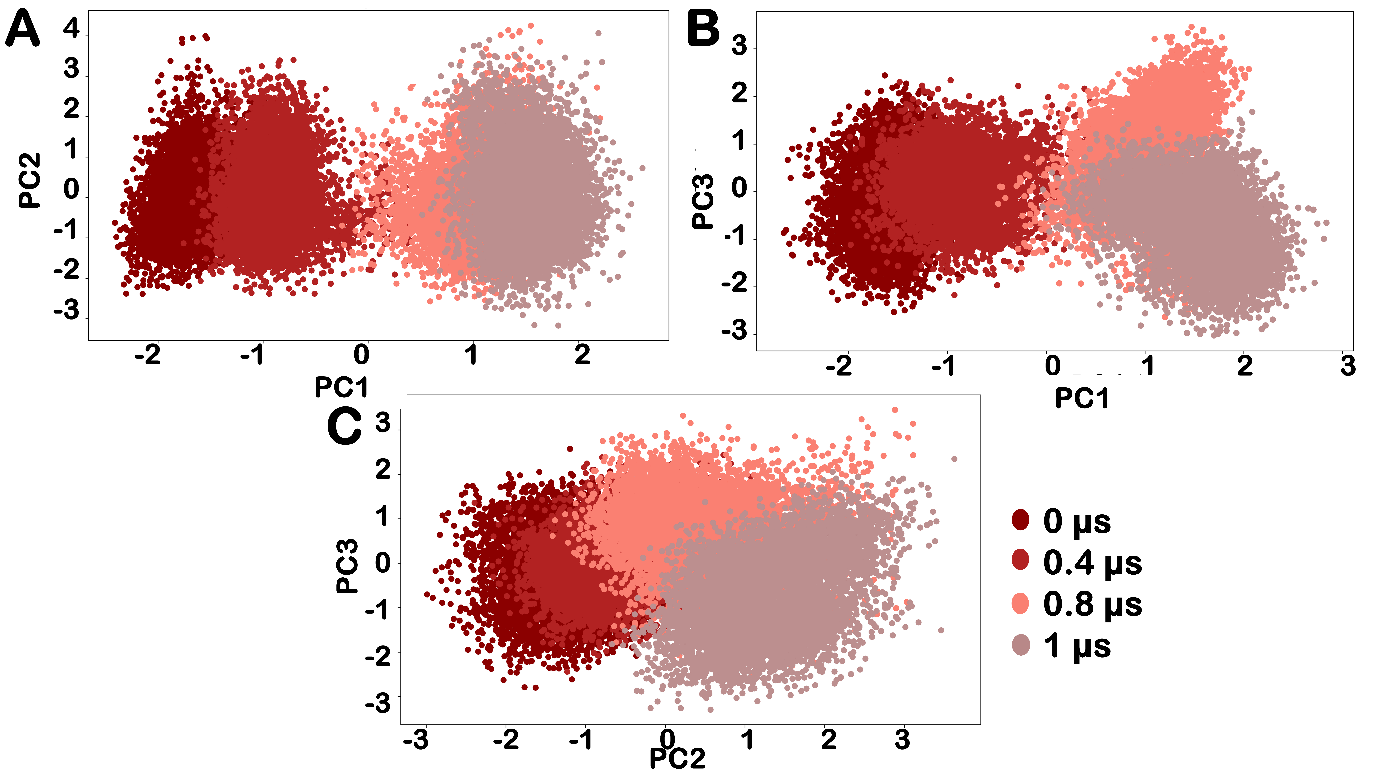


**Supplementary Figure 17: Principal component analysis (PCA) of gRNA-DNA hybrid bound with K949A mutant of RVR variant of AsCpf1 protein, with colour variants representing different time frames in each plot.** The 2-D plot represents the Stability in hybrid. All three principal components shown in two dimensions represent the essential conformational dynamics of gRNA-DNA hybrid complexed with K949A mutant of RVR variant of AsCpf1.


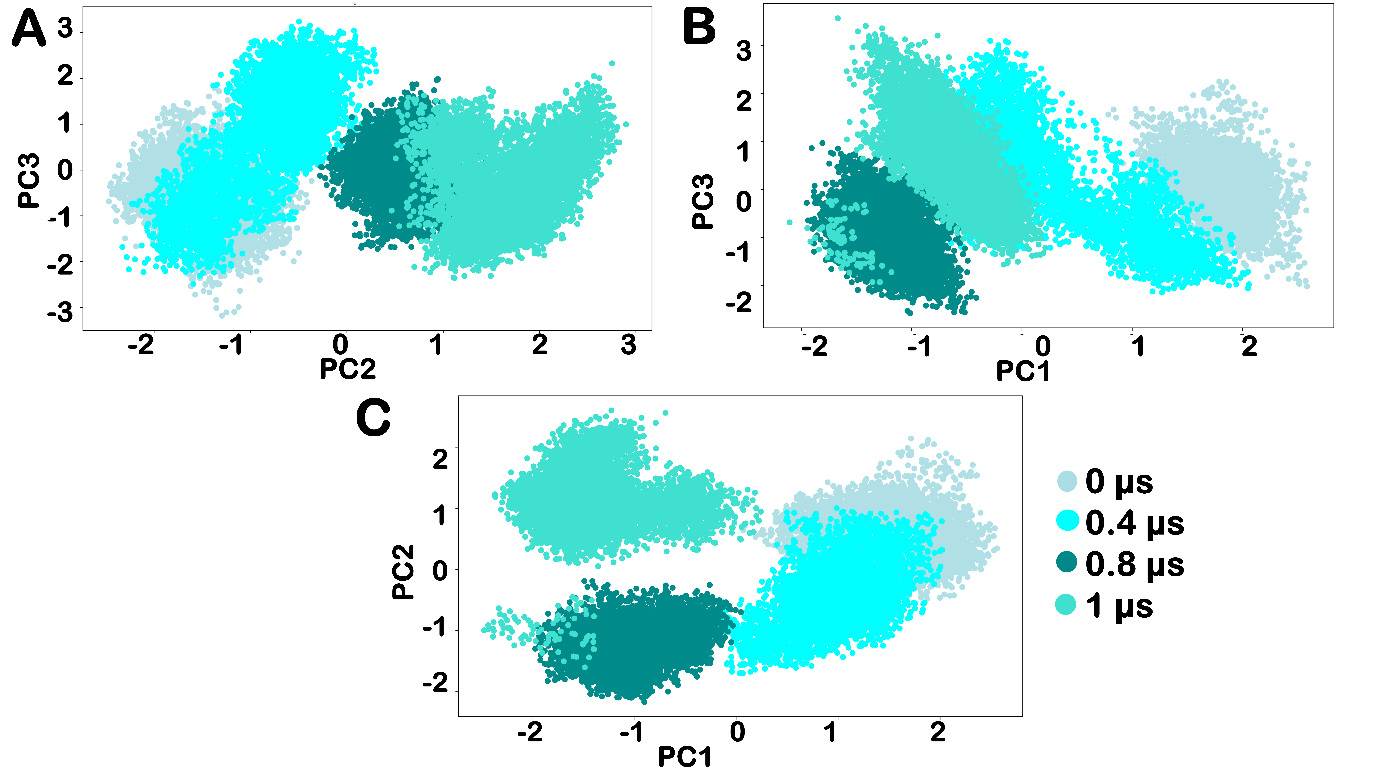


**Supplementary Figure 18: Principal component analysis (PCA) of Wildtype AsCpf1 protein, with colour variants representing different time frames in each plot.** The 2-D plot represents the flexibility and conformational dynamics of complexes. All three principal components shown in two dimensions represent the essential conformational dynamics of Wildtype AsCpf1.


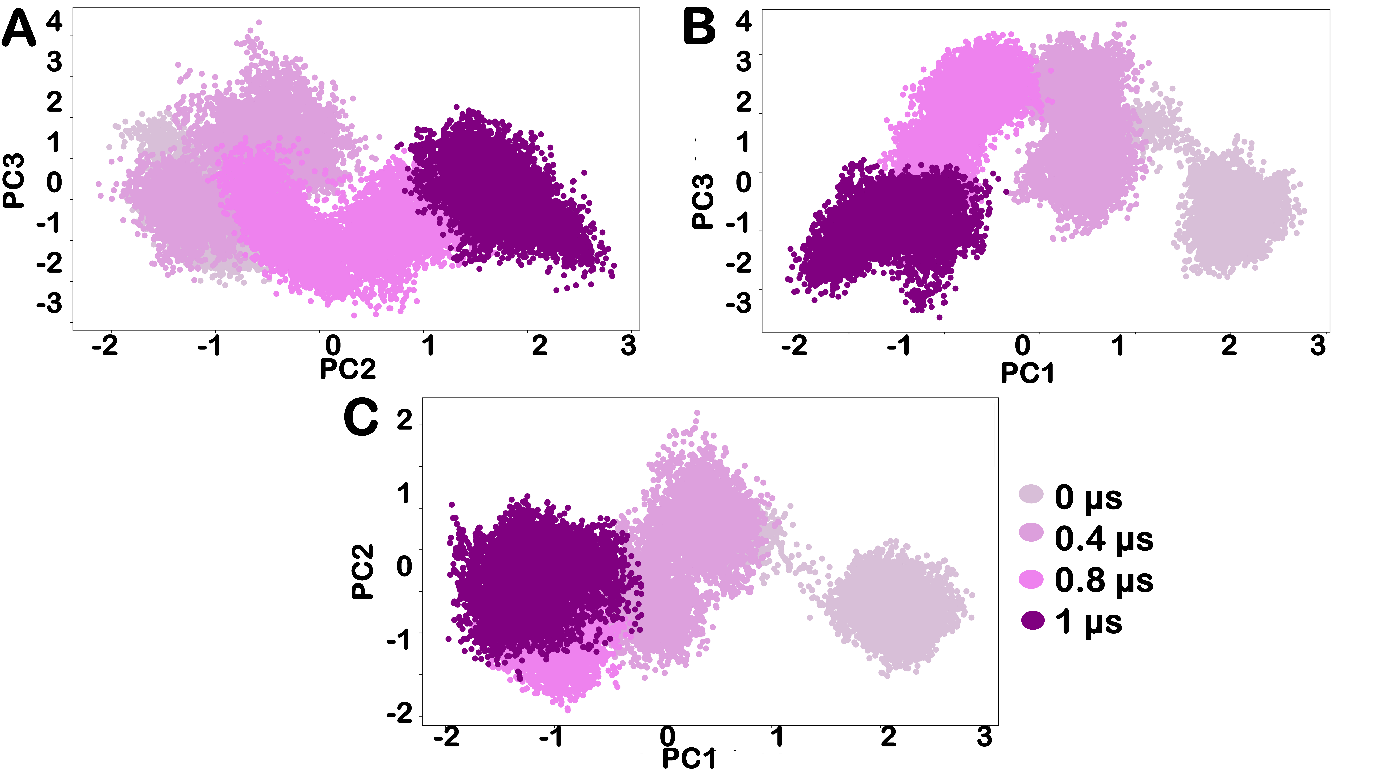


**Supplementary Figure 19: Principal component analysis (PCA) of RR variant of AsCpf1 protein, with colour variants representing different time frames in each plot.** The 2-D plot represents the flexibility and conformational dynamics of complexes. All three principal components shown in two dimensions represent the essential conformational dynamics of RR variant of AsCpf1.


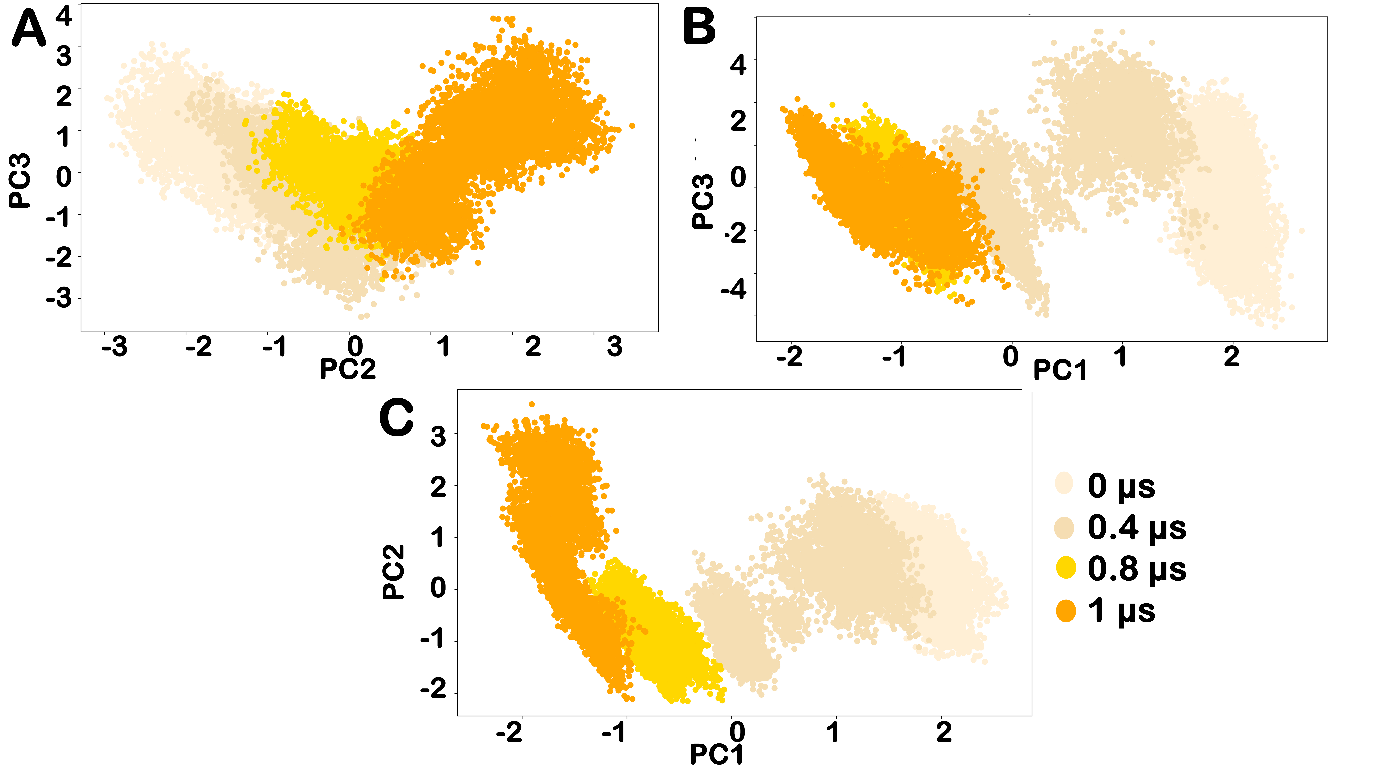


**Supplementary Figure 20: Principal component analysis (PCA) of RVR variant of AsCpf1 protein, with colour variants representing different time frames in each plot.** The 2-D plot represents the flexibility and conformational dynamics of complexes. All three principal components shown in two dimensions represent the essential conformational dynamics of RVR variant of AsCpf1.


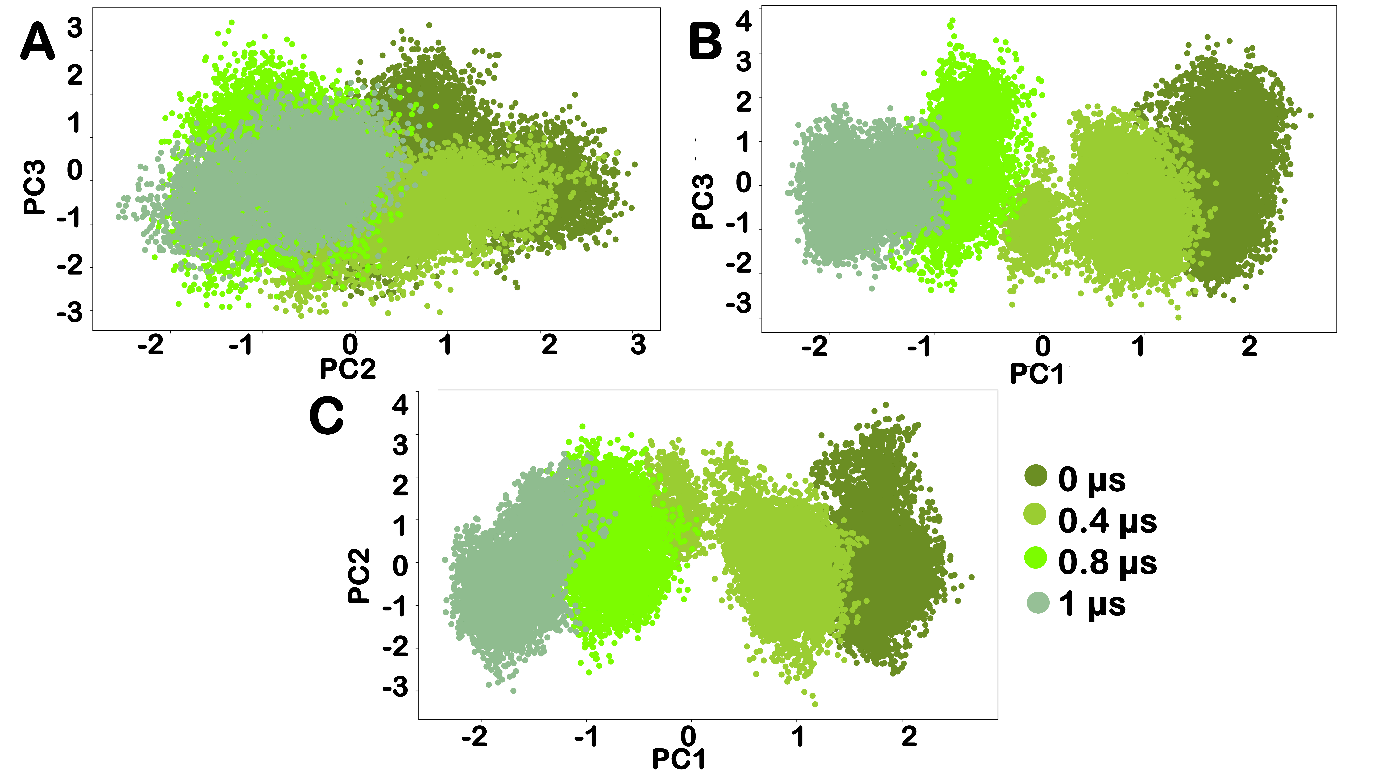


**Supplementary Figure 21: Principal component analysis (PCA) of K949A mutant of RR variant of AsCpf1 protein, with colour variants representing different time frames in each plot.** The 2-D plot represents the flexibility and conformational dynamics of complexes. All three principal components shown in two dimensions represent the essential conformational dynamics of K949A mutant of RR variant of AsCpf1.


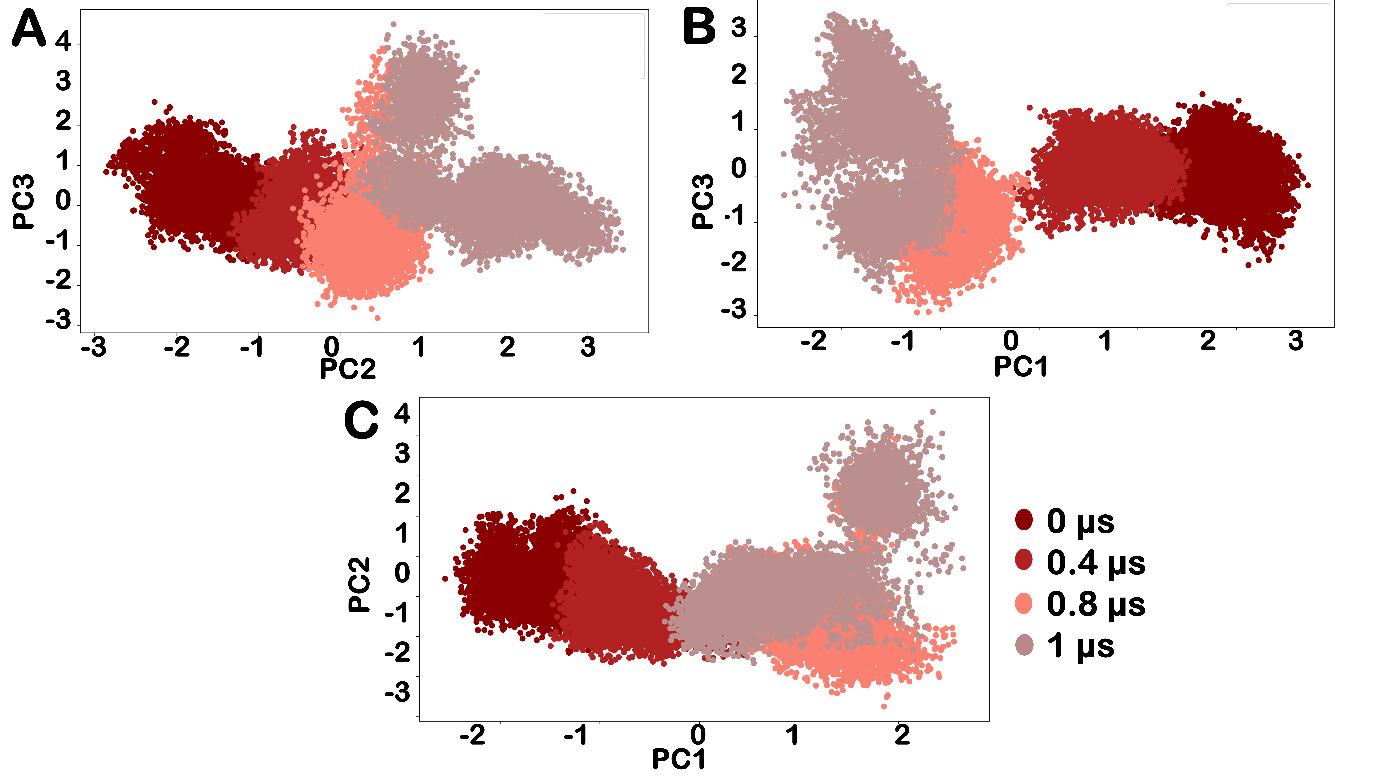


**Supplementary Figure 22: Principal component analysis (PCA) of K949A mutant of RVR variant of AsCpf1 protein, with colour variants representing different time frames in each plot.** The 2-D plot represents the flexibility and conformational dynamics of complexes. All three principal components shown in two dimensions represent the essential conformational dynamics of K949A mutant of RVR variant of AsCpf1.


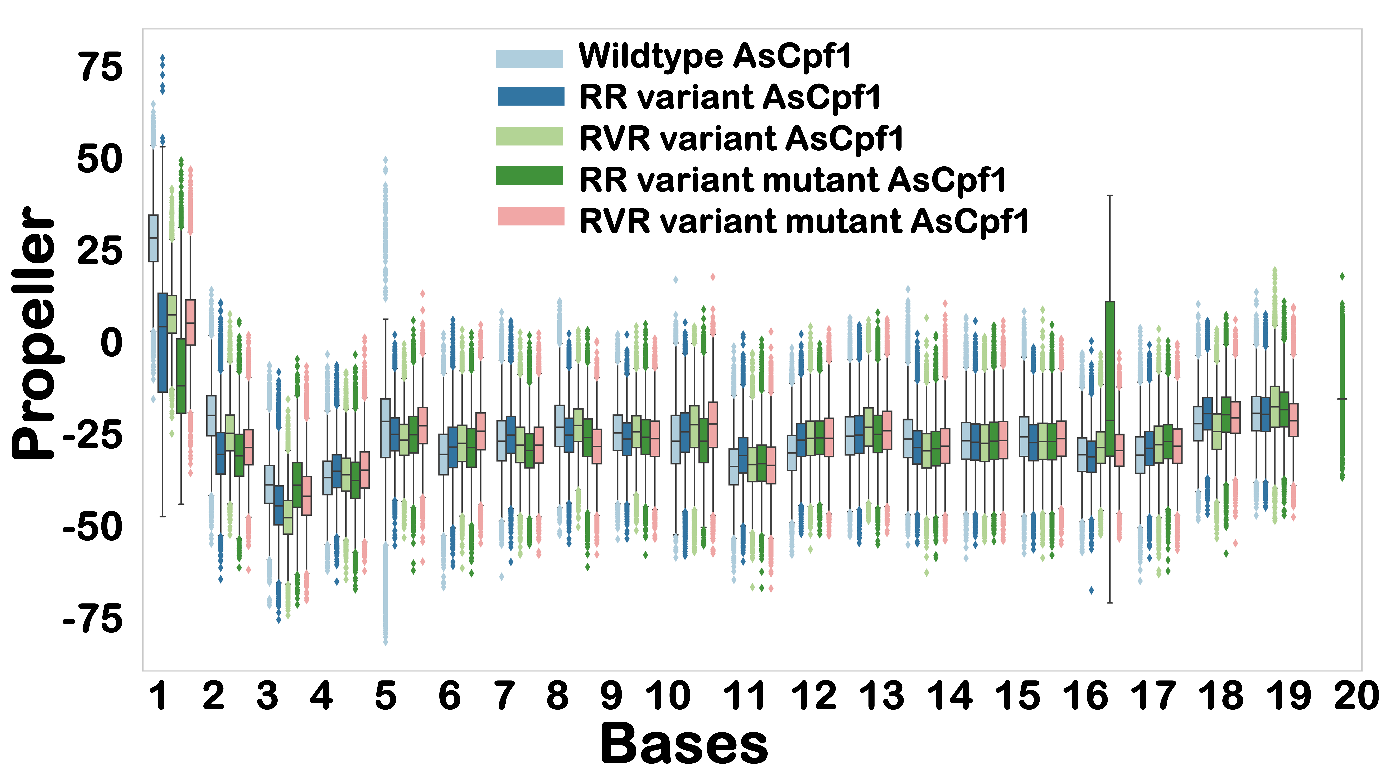


**Supplementary Figure 23: The representation of structural distortion in the base pair structural parameter “Propeller” in gRNA-DNA hybrid over an extended time frame of 1µs.** Structural base pair fluctuations in the gRNA-DNA hybrid when bound to Wildtype, RR, RVR, mutant of RR, and RVR variant for each position in terms of Propeller are represented. The generated boxplot has the median in the box and the 25th and 75th quartiles as whiskers in the plot. The data points outside the whiskers are outliers and represent the prominent structural distortions in the respective heteroduplexes.


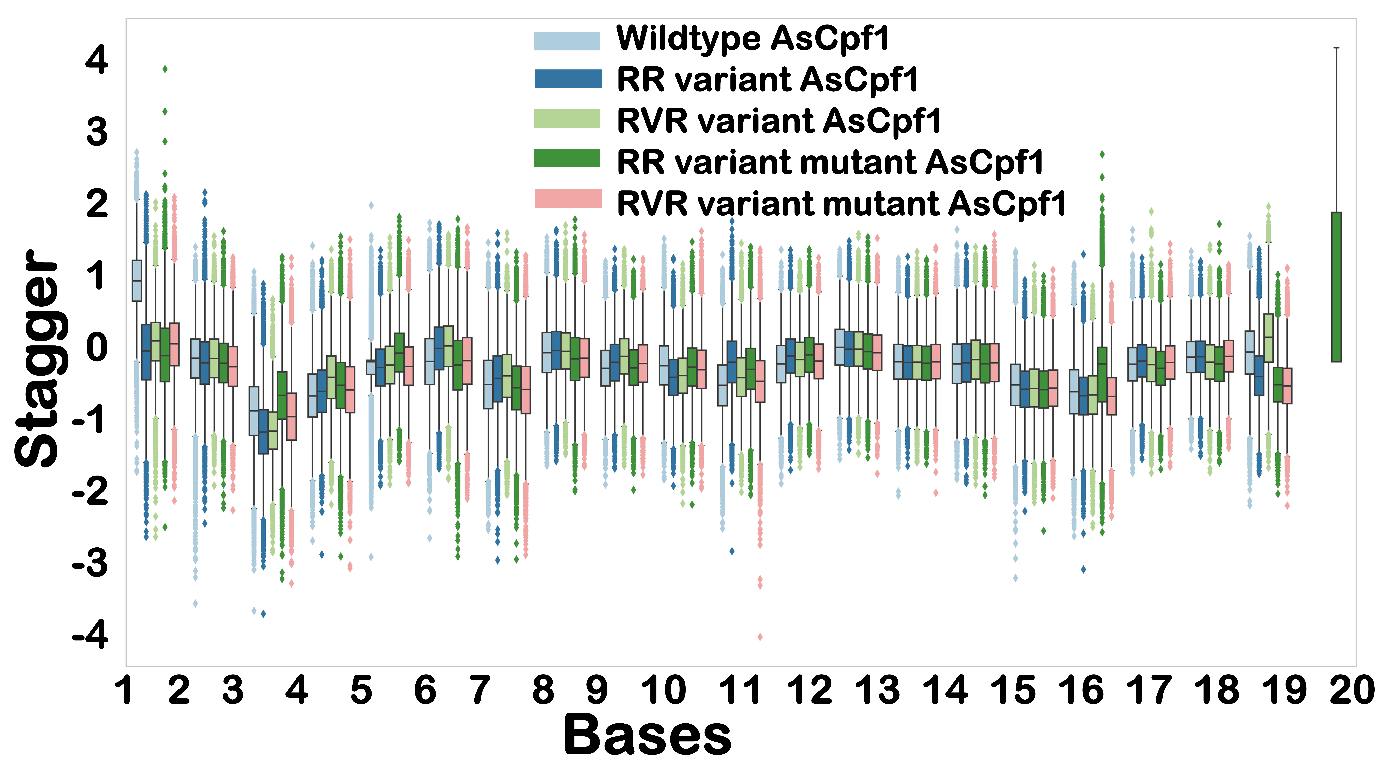


**Supplementary Figure 24: The representation of structural distortion in the base pair structural parameter “Stagger” in gRNA-DNA hybrid over an extended time frame of 1µs.** Structural base pair fluctuations in the gRNA-DNA hybrid when bound to Wildtype, RR, RVR, mutant of RR, and RVR variant for each position in terms of Stagger are represented. The generated boxplot has the median in the box and the 25th and 75th quartiles as whiskers in the plot. The data points outside the whiskers are outliers and represent the prominent structural distortions in the respective heteroduplexes.
